# AI‐enhanced Centiloid quantification of amyloid PET images

**DOI:** 10.1002/alz.71162

**Published:** 2026-02-11

**Authors:** Pierrick Bourgeat, Jurgen Fripp, Leo Lebrat, Ying Xia, Azadeh Feizpour, Timothy Cox, Georgios Zisis, Ashley Gillman, Manu S. Goyal, Duygu Tosun, Tammie LS. Benzinger, Pamela LaMontagne, Michael Breakspear, Michelle K. Lupton, Cathy Short, Robert Adam, Joanne S. Robertson, Reisa Sperling, Sid E. O'Bryant, Sterling C. Johnson, Clifford R. Jack Jr, Christopher G. Schwarz, Frederik Barkhof, Gill Farrar, Ariane Bollack, Lyduine E. Collij, Susan Landau, Robert Koeppe, for the Alzheimer's Disease Neuroimaging Initiative, OASIS3, the A4/LEARN Study Team, the AMYPAD consortium, The Health and Aging Brain Study (HABS‐HD) Study Team, Mayo Clinic Study of Aging, WRAP, ADNI‐DOD, PISA, ADNeT, the AIBL research group, John C. Morris, Michael W. Weiner, Victor L. Villemagne, Colin L. Masters, Christopher C. Rowe, Vincent Dore

**Affiliations:** ^1^ Australian eHealth Research Centre CSIRO Health and Biosecurity Brisbane QLD Australia; ^2^ School of Electrical Engineering & Robotics, Faculty of Engineering Queensland University of Technology Brisbane QLD Australia; ^3^ Department of Molecular Imaging & Therapy Austin Health Melbourne VIC Australia; ^4^ The Florey Institute of Neuroscience and Mental Health University of Melbourne, Parkville Melbourne VIC Australia; ^5^ Mallinckrodt Institute of Radiology Washington University School of Medicine St Louis Missouri USA; ^6^ Department of Neurology Washington University School of Medicine St. Louis Missouri USA; ^7^ Department of Radiology and Biomedical Imaging University of California San Francisco California USA; ^8^ School of Psychological Sciences University of Newcastle Newcastle NSW Australia; ^9^ Brain and Mental Health QIMR Berghofer Medical Research Institute Brisbane QLD Australia; ^10^ School of Biomedical Sciences Faculty of Medicine University of Queensland Brisbane Queensland Australia; ^11^ School of Biomedical Sciences Faculty of Health Queensland University of Technology Brisbane Queensland Australia; ^12^ Central Adelaide Local Health Network Memory Service and Memory Clinical Trials Adelaide SA Australia; ^13^ Centre for Health Services Research The University of Queensland Brisbane QLD Australia; ^14^ Harvard Medical School Brigham and Women's Hospital Massachusetts General Hospital Boston Massachusetts USA; ^15^ Institute for Translational Research University of North Texas Health Science Center Fort Worth Texas USA; ^16^ Alzheimer's Disease Research Center University of Wisconsin‐Madison Madison Wisconsin USA; ^17^ Department of Radiology Mayo Clinic Rochester Minnesota USA; ^18^ Brain Imaging, Amsterdam Neuroscience Amsterdam the Netherlands; ^19^ Radiology & Nuclear Medicine Vrije Universiteit Amsterdam, Amsterdam UMC location VUmc Amsterdam The Netherlands; ^20^ Queen Square Institute of Neurology and Centre for Medical Image Computing University College London London UK; ^21^ GE HealthCare Chalfont St Giles UK; ^22^ Department of Medical Physics and Biomedical Engineering University College London London UK; ^23^ Department of Neuroscience University of California Berkeley California USA; ^24^ Division of Nuclear Medicine Department of Radiology University of Michigan Ann Arbor Michigan USA; ^25^ San Francisco Veterans Affairs Medical Center San Francisco California USA; ^26^ Department of Psychiatry The University of Pittsburgh Pittsburgh Pennsylvania USA

**Keywords:** Alzheimer's disease, amyloid positron emission tomography, Centiloid, deep Learning, longitudinal analysis

## Abstract

**INTRODUCTION:**

The Centiloid scale is the standard for amyloid (Aβ) PET quantification in research and clinical settings. However, variability between tracers and scanners remains a challenge.

**METHODS:**

This study introduces DeepSUVR, a deep learning method to correct Centiloid quantification, by penalizing implausible longitudinal trajectories during training. The model was trained using data from 2,129 participants (7,149 Aβ positron emission tomography [PET] scans) in the Australian Imaging, Biomarkers and Lifestyle Study of ageing (AIBL)/Alzheimer's Disease Neuroimaging Initiative (ADNI) and validated using 15,807 Aβ PET scans from 10,543 participants across 10 external datasets.

**RESULTS:**

DeepSUVR increased correlation between tracers, and reduced variability in the Aß‐negatives. It showed significantly stronger association with cognition, visual reads, neuropathology, and increased longitudinal consistency between studies. DeepSUVR also increased the effect size for detecting small treatment related slowing of amyloid accumulation in the A4 study.

**DISCUSSION:**

DeepSUVR substantially advances Aβ PET quantification, outperforming all standard approaches, which is particularly important for clinical decision making and to detect subtle or early changes in Aβ.

**Highlights:**

- Novel artificial intelligence (AI)‐method that penalizes biologically implausible longitudinal trajectories, enabling the model to learn standardized uptake value ratios (SUVR) correction factors without requiring longitudinal data at inference time.
- Improves Centiloid consistency across tracers and studies, significantly enhancing cross‐sectional and longitudinal amyloid positron emission tomography (PET) quantification.
- DeepSUVR‐derived Centiloids show stronger associations with cognition, visual reads, and neuropathology.
- Longitudinal variability is reduced three‐ to five‐fold, enabling more reliable tracking of amyloid accumulation and better detection of treatment effects.
- Novel reference and target masks derived from DeepSUVR replicate most of the model's performance, offering a practical alternative for integration into existing pipelines.

## 1 BACKGROUND

The Centiloid scale was developed to unify the quantification derived from all β‐amyloid (Aβ) positron emission tomography (PET) tracers onto a single, standardized scale.^1^ It has been widely adopted as the default quantification scale in Aβ PET research and serves as a secondary end‐point in anti‐Aβ clinical trials.^2^, ^3^ Accurate Centiloid quantification is crucial for: (1) reducing diagnosis uncertainty; (2) informing patient inclusion criteria for anti‐Aβ disease‐modifying therapies; (3) accurately quantifying Aβ clearance during therapeutic interventions; and (4) supporting the identification of early or emerging Aβ pathology.^4^ While the Centiloid framework has significantly advanced the standardization of Aβ quantification across diverse studies and tracers,^5^ its current standard implementation assumes that the proposed reference and target masks are universally optimal. This may overlook variability introduced by different PET tracers, scanners or image reconstruction parameters, as seen in head‐to‐head and longitudinal studies.^6^, ^7^

Prior to the Centiloid, tracer‐specific reference and target regions for calculating the standardized uptake value ratio (SUVR) were developed. While the target regions were similar across tracers, the optimal reference region varied significantly: cerebellum cortex (Cb) for ^11^C‐PiB (PIB),^8^ ^18^F‐Florbetaben (FBB),^9^ and ^18^F‐NAV4694 (NAV),^10^ pons for ^18^F‐Flutemetamol (FMM)^11^ and whole cerebellum (WCb) for ^18^F‐Florbetapir (FBP),^12^ though a composite reference (eroded subcortical white matter [WM] mask+WCb) was found to be superior for both longitudinal^13^ and cross‐sectional FBP analyses.^6^ Finally, while the Centiloid neocortical mask was defined using a data‐driven approach, the reference mask was anatomically delineated, making it more susceptible to subjectivity and potential suboptimality.

Data‐driven approaches such as Aβ load,^14^ Aβ index,^15^ and non‐negative matrix factorization (NMF)^16^ decompose the image into specific and non‐specific components, potentially reducing the influence of the reference region. A recent comparison showed potential for reducing the number of participants in simulated prevention trial scenarios.^17^ However, broader adoption of these methods is hindered by limited availability, limited validation, and reliance on fixed preprocessing pipelines.

Deep learning (DL) strategies have also been explored, typically either replacing the conventional spatial normalization to a template^18^ or directly estimating the Centiloid^19^ or amyloid positivity^20^ from raw PET images. However, since these methods emulate the standard pipeline or are trained to reproduce its quantification, they inherit the same limitations.

This study hypothesizes that sub‐optimal reference and/or target regions are the main sources of variability in longitudinal Centiloid measurements. Given that SUVR is a ratio of tracer retention in these two regions, a SUVR correction factor could mitigate variability from either region. We further hypothesize that this correction factor could be estimated by penalizing deviations from the expected longitudinal trajectory of amyloid accumulation. The natural history curve for Aβ accumulation, initially conceptualized by Jack and colleagues^21^ has been independently characterized by multiple groups.^22^, ^23^, ^24^, ^25^, ^26^, ^27^ This model implies that an individual's current Centiloid value is predictive of their future Centiloid changes. Within a machine learning framework, this curve can serve as a temporal prior, with deviations from the curve penalized during training. We further hypothesize that the resulting DL‐corrected SUVR can be used to define new, data‐driven reference, and target masks for both DL network explainability and simplified implementation in existing pipelines.

We developed and trained a DL model (DeepSUVR) using observational longitudinal data to predict a SUVR correction factor, which once transformed into Centiloid, minimizes deviations from the established amyloid natural history curve. DeepSUVR was trained using five‐fold cross‐validation on the combined Australian Imaging, Biomarkers, and Lifestyle Study of ageing AIBL and Alzheimer's Disease Neuroimaging Initiative (ADNI) dataset. It was subsequently validated using data from three cross‐sectional and seven longitudinal cohorts. The corrected SUVRs were then used to generate novel reference and target masks. The resulting Centiloids are compared against the Standard Centiloid (CL_Std_), Centiloids computed using the composite reference region for FBP (CL_Comp_), and our previously proposed NMF approach^16^ (CL_NMF_), across both the training and testing cohorts.

The DeepSUVR model and derived masks are freely available for non‐commercial use (https://github.com/csiro/DeepSUVR).

## 2 METHODS

### 2.1 Data

Data used in this study combined 12 imaging studies in Alzheimer's disease: the AIBL,^28^ ADNI,^29^ the Open Access Series of Imaging Studies [OASIS3],^30^ the Anti‐Amyloid Treatment in Asymptomatic Alzheimer's [A4] Study, and Longitudinal Evaluation of Amyloid Risk and Neurodegeneration [LEARN] Study,^31^ the Amyloid imaging to prevent Alzheimer's disease [AMYPAD],^32^, ^33^, ^34^ the Mayo Clinic Study of Aging [MCSA],^35^ the Health and Aging Brain Study: Health Disparities [HABS‐HD],^36^ the Dallas Lifespan Brain Study [DLBS],^37^ the Wisconsin Registry for Alzheimer's Prevention [WRAP],^38^ the Department of Defense Alzheimer's Disease Neuroimaging Initiative [ADNI‐DOD],^39^ the Prospective Imaging Study of Ageing [PISA],^40^ and the Alzheimer's Disease Network [ADNeT].^41^

Data used in the preparation of this article were partly obtained from the ADNI database (adni.loni.usc.edu). The ADNI was launched in 2003 as a public‐private partnership, led by Principal Investigator Michael W. Weiner, MD. The primary goal of ADNI has been to test whether serial magnetic resonance imaging (MRI), PET, other biological markers, and clinical and neuropsychological assessment can be combined to measure the progression of mild cognitive impairment (MCI) and early Alzheimer's disease (AD). For up‐to‐date information, see http://www.adni‐info.org.

**RESEARCH IN CONTEXT**

1. **Systematic review**: We reviewed the literature on β‐amyloid (Aβ) positron emission tomography (PET) quantification methods, with a focus on the Centiloid scale and its implementation across different tracers. While the Centiloid scale is widely adopted, its reliance on fixed reference and target regions introduces variability across tracers, scanners, and studies.
2. **Interpretation**: This study introduces DeepSUVR, a deep learning model trained to correct standardized uptake value ratios (SUVRs) by penalizing biologically implausible longitudinal trajectories. DeepSUVR improves inter‐tracer agreement, reduces variability in Aβ‐negative scans across tracers and studies, and increase associations with cognition, visual reads, and neuropathology. It also enables the derivation of new reference and target masks for broader use in existing pipelines.
3. **Future directions**: Future work will focus on validating this approach in clinical trial settings and exploring its utility in early detection and disease progression modeling.

### 2.2 Visual reads

All visual reads were provided by the parent studies, and the respective variable is provided. In A4, 92 scans were read by two readers local to the imaging site. For 12 scans where the reads were discordant, the consensus between the two readers was used [variable “consensus” in imaging_PET_VA.csv]. The remaining 1680 scans were read by a single reader [variable “elig_vi_1″ in imaging_PET_VA.csv]. In ADNI and ADNI‐DOD, a single reader performed an initial visual read. For challenging cases and in cases where the visual read and the quantification were discordant, at least three readers performed a consensus review of the scans. When available, the consensus review was used [Variable “CONSENSRES” in AMYREAD.csv (ADNI); Variable “FINALREAD” in UCSF_AV45_VISUALREAD.csv (ADNI‐DOD)]. Otherwise, the initial read was used [Variable “OUTCOME” in AMYREAD.csv (ADNI)]. In HABS‐HD, the PET images were interpreted by licensed fellowship trained radiologists specializing in Nuclear Neuro”based imaging recognizing Amyloid and Tau positivity [Variable “ClinicalRead_Amyloid_Positivity” in HD_Visit_*_Release_5_FINAL.csv]. In AMYPAD, images were rated, together with a T1‐weighted MRI scan or CT scan, as either positive (binding in one or more cortical brain region unilaterally, or striatum in the case of FMM) or negative (predominantly white matter uptake)^42^ [Variable “pet_vr_classification” in AMYPAD_PNHS_PET_Variables_202305.csv].

### 2.3 Preprocessing

The preprocessing of the PET images was previously described,^6^ except that in this work, there was no smoothing to a uniform 8 mm full width half‐maximum (FWHM) resolution, and only “raw” scans in their native resolution were used. Briefly, all PET images were co‐registered to their matching T1W MRI. The T1W MRIs were spatially normalized (non‐linearly) to the MNI template with SPM8. The transform was then applied to the raw PET images. An outline of the preprocessing steps is provided in supplementary Figure 1. All the spatial normalizations were visually checked and failed registrations corrected using different parameters for the initial rigid/affine registration. The SPM8 probabilistic Montreal Neurological Institute (MNI) ‐space brain mask, thresholded at 0.5 is applied to all spatially normalized images to remove the contribution of noncortical voxels and reduce the memory footprint when training the model.

### 2.4 Baseline methods

In addition to the standard Centiloid method, two other Centiloid quantification methods are used to define baseline comparisons.

‐ The composite reference region, which includes subcortical white matter as well as the whole cerebellum and that was proposed to improve the ability of measuring longitudinal changes with FBP.^13^ To minimize the contribution from voxels with the partial volume effects at the grey‐white matter boundary, the white matter segmentation from SPM is first smoothed using an 8 mm Gaussian kernel and then thresholded at 70% of its maximum to erode the white matter mask away from grey matter, before being combined with the Centiloid whole cerebellum mask. In subsequent experiments, the composite reference region is only used for FBP, with the Standard CL used for the other tracers.
‐ The non‐negative matrix factorization (NMF) which we have previously proposed to improve tracer harmonisation^6^, ^16^ and which was recently shown to be one of the most sensitive measure of changes in amyloid accumulation rate.^17^ Briefly, all PIB scans from the GAAIN calibration dataset were decomposed using a six components NMF, where the coefficients of the first component are used as a proxy of Aβ burden, and calibrated into Centiloids. For each F18 tracers, a new NMF basis was computed so that the resulting coefficients of the first component would match those of the corresponding PIB scans in the GAAIN calibration dataset. This allows each tracer's NMF decomposition to model differences between tracers, ensuring comparable quantification.

### 2.5 Model

In the proposed model, DeepSUVR estimates a SUVR correction factor (*CF*) given a spatially and SUVR normalized image (where the image intensity is divided by the uptake in the whole cerebellum) and the corresponding tracer used. The network is based on our previous work^43^ where it was used to predict an intensity rescaling of MR images to improve tumor segmentation. However, the loss function and training strategy in this work are different since no longitudinal information was used in our original work. The network is illustrated in Figure S2. The model includes four convolutional blocks, each with a 3D convolution (with increasing channel numbers from 16 to 128, kernel size = 4 and stride = 4), followed by instance normalization and LeakyReLU. The output of the last convolutional block is flattened and concatenated with the tracer information, which is encoded as a one‐hot vector of length 5. The first two fully connected blocks include a dropout with a rate of 0.5, a fully connected layer, batch normalization and Tanh. The last layer includes a fully connected layer followed by a sigmoid to generate the *CF*. The sigmoid ensures that *CF* is within the [0–1] range. Finally, to allow a full range of SUVR corrections, *CF* is multiplied by 2 to be constrained to the [0–2] positive range. This range was chosen to provide the model with sufficient flexibility to both down‐correct (*CF* < 1) and up‐correct (*CF* > 1) the initial SUVR, while preventing extreme, unbounded adjustments.

### 2.6 Training strategy

The training set was split into five subsets for a five‐folds cross‐validation. The subsets were balanced so that each fold had similar distribution of tracers, number of visits and Centiloid values and each participant could only belong to a single subset. The other 10 datasets were kept as independent test sets.

For each participant of the combined AIBL/ADNI dataset, pairs of PET images which were acquired at least 3 months apart, but no more than 3.5 years apart were selected. Shorter timeframes tend to lead to high yearly rates of change, even when the absolute change is small, and could therefore have a disproportionate influence on the longitudinal loss function, and hinder convergence. Longer timeframes can potentially bias the estimate of the Aβ accumulation curve due to its non‐linear nature, especially if a participant's amyloid trajectory accelerates significantly during the interval, where a linear estimate would not accurately represent the true mean CL and mean rate of accumulation. When three or more images were available within that timeframe, all permutations were considered (i.e. for three visits within 3.5 years, we would include the pairs1^–^2, 2^–^3, and1^–^3). It is important to note that this is a training constraint designed to ensure the model learns from reliable estimates of annual change and is not a limitation of DeepSUVR at inference time, which can be run on any single scan or pair of scans regardless of the time interval.

**FIGURE 1:**
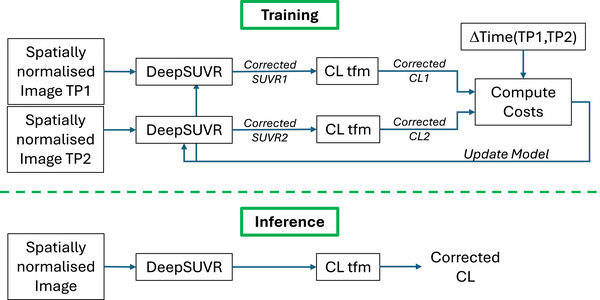
Framework used for training (top) and inference (bottom) using DeepSUVR. While training requires pairs of images from the same participant, inference is run on single images. CL tfm refers to the Centiloid transformation, which is the tracer‐specific linear equation that converts a standardized uptake value ratio (SUVR) value into a Centiloid value.

The training strategy for DeepSUVR is illustrated in Figure 1. For each pair, both images and their respective tracer information were run in succession through the DeepSUVR model during the same iteration, generating two *CFs*. The order in which the images were run through the network was randomized to ensure that it could not be learnt.

Data augmentation included random rotations (max 5°) and nonrigid deformation (sigma = 20 voxels, magnitude = 50 voxels) to model small errors in spatial normalization. It also included random Gaussian smoothing to model differences in PET resolution from different scanners. The smoothness was constrained based on each scanner point‐spread function (PSF), as measured using a Hoffman phantom on each scanner of the training set as per the methodology of Joshi *et al.*,^44^ so that for each scan of a given scanner, the smoothing was constrained to the FWHM range [PSF_Scanner_, 8 mm]. The data augmentation was implemented using the MONAI framework.^45^

While longitudinal data are required to train the model, the model only requires a single image for inference. This means that DeepSUVR can be used to correct both longitudinal and cross‐sectional studies.

### 2.7 Loss function

For each pair, the loss function takes as input the standard SUVRs and their corresponding *CF* as estimated by the DeepSUVR model. It first computes the corrected SUVR as CF*SUVR and converts it into a corrected Centiloid using the standard and previously published Centiloid transforms.^46^ Using those corrected Centiloid values, four loss functions are computed.

**FIGURE 2:**
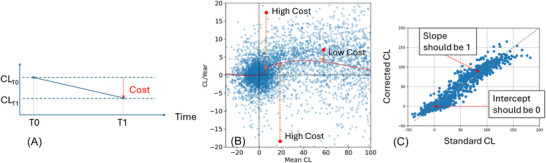
The penalties in the loss functions used to constrain the model: (A) for each pair of TPs, penalize the Centiloid decreasing over time; (B) penalize the distance to the population curve of Mean CL vs. Rate of change, with points further from the curve incurring a high cost, or penalty, compared to those closer to the curve; (C) for each batch, penalize the regression line of the standard CL vs. corrected CL when it deviates from slope = 1 and intercept = 0.

- *L*_d_ – Penalizes the Centiloid decreasing over time (Figure 2a): In an observational study, the Centiloid should always remain constant or increase over time but should not be decreasing. Therefore, we penalize decrease in Centiloids, with the loss being directly proportional to the amount of decrease. If the Centiloid remains constant or increases, then the loss is set to 0. Given two corrected Centiloid values *CL*_T0_ and *CL*_T1_ acquired at time T0 and T1 with T1 > T0, the loss is defined as:
$$L_{d}=\max\left(0,CL_{T0}-CL_{T1}\right)$$
- *L*_c_ – Penalizes deviations from the expected rate of change curve (Figure 2b): As a pre‐requisite, we need to first establish the reference curve that describes the relationship between the Centiloid value and its rate of change. This is performed by first computing for each pair of timepoints in the combined AIBL/ADNI dataset the mean Centiloid and the rate of change (in CL/Year) using the standard Centiloid quantification. To reduce variability due to the use of different tracers, this is restricted to PIB scans only. A locally weighted scatterplot smoothing (lowess) *f*_c_ with a smoothing parameter = 0.2 is fitted and used as a proxy to describe the expected rate of change for a given Centiloid value (Figure 2b). This fit was performed once on the entire AIBL/ADNI PIB training dataset to establish a single, static reference curve that was used for all training folds. The lowess smoothing parameter was chosen empirically to provide a robust fit to the general shape of the accumulation curve without overfitting to noise and local fluctuations in the standard Centiloid data. Using the corrected Centiloid values, we compute the mean and rate of change. The absolute difference between the actual rate of change and the expected rate of change given the mean corrected Centiloid value is used to penalise unexpected changes in Centiloids over time, so that
$$L_{c}=\left|\left(CL_{T1}-CL_{T0}\right)/\left(T1-T0\right)-f_{c}\left((CL_{T1}+CL_{T0})/2\right)\right|$$
- *L*_s_ *and L*_i_ – Penalizes the Centiloid deviating from the uncorrected Centiloid value (Figure 2c). Without extra constraints, there is no penalty for the model over or under‐correcting the Centiloids. In the extreme case, it could collapse all correction factors to 0. To ensure that the corrected Centiloids still represent meaningful quantities, we compute a regression line between the corrected and uncorrected Centiloid value for the entire batch, with the constraint that the slope should be 1 and the intercept should be 0, therefore ensuring that the Corrected Centiloids are still meaningfully associated with the uncorrected Centiloids. *L*_s_ and *L*_i_ should be regarded as batch‐level regularization terms to prevent scale drift and model collapse, rather than as a direct regression target. Given the slope *s* and the intercept *i* of the regression line between the corrected and uncorrected Centiloids, the losses are defined as
$$L_{s}=|s-1|andL_{i}=\left|i\right|$$
- The four losses are combined in a single loss with weights α, β, γ that were set empirically as a compromise between getting the best fit to the curve while minimizing deviations from the standard Centiloids. This was performed using first a coarse grid search, followed by a refinement of α to find a suitable balance between optimizing longitudinal consistency and regularizing against scale drift:
$$L=L_{d}+\alpha L_{c}+\beta L_{s}+\gamma L_{i}$$

### 2.8 Training

The model was trained on a H100 Nvidia card using a batch size of 128. The input images had a size of 91 × 109 × 91 with a 2 mm isotropic voxel spacing. The weights for each loss were set to *α* = 0.2, *β* = 1, *γ* = 0.01. The model was trained with early stopping, monitoring the validation loss. Training was stopped when the validation loss did not improve for 20 consecutive epochs. To reduce the risk of the model converging to a suboptimal local minimum, for each fold, the model was trained using five different weight initializations and dataloader shuffling, and the model with the highest Spearman rank correlation between the mean *CL* and rate of *CL* change in the validation set was selected as the best model.

### 2.9 Generating new Centiloid masks

Deep learning models are often known for being black boxes, and their interpretability can be extremely challenging. Since the model provides an overall correction, which cannot be directly attributed to a change in the target or reference region, traditional explainability methods, such as GradCAM are not suitable, as we cannot relate activation maps to specific correction to the reference or target region. Instead, we reformulate the explainability problem with the hypothesis that the corrections provided by DeepSUVR reflect changes in either the Centiloid reference or target mask, or both. To validate this hypothesis, we here aim to optimize new reference and target masks that maximize the correlation with the corrected SUVR values. This is formulated as an optimization problem with a new reference and target mask being optimized across all tracers using the AIBL+ADNI training dataset so that the resulting SUVR maximize the Pearson correlation coefficient with the DeepSUVR‐corrected SUVRs. The new masks are derived using a direct optimization procedure, where the voxel intensities of the reference and target masks are treated as learnable parameters. An overview of the optimization approach is provided in Figure S3. A gradient descent algorithm is used to iteratively update these voxel values to maximize the following two losses:

- *L*_p_ Penalizes low Pearson correlation coefficient. Using the updated masks, new SUVRs can be computed across the entire dataset. The correlation between these SUVRs and the Corrected SUVR obtained by DeepSUVR is used to compute the Pearson coefficient R^2^. The resulting loss is defined as:
$$L_{p}=1-R^{2}$$
- *L*_b_ Promote binary masks. The two masks being optimized, need to have continuous values to ensure that they can be derived throughout the optimization process. To force the mask *M* towards binarity, values not being either 1 or 0 are penalized. Additionally, to normalize the loss for different resolutions, the loss is normalized by the number of nonzeros voxels B in the brain mask as follow:
$$L_{b}=\sum \left(0.5-|M-0.5|\right)/B$$
- The Pearson loss *L*_p_ is computed for each tracer separately, and the average across the five tracers is used in the total loss. The binarity loss *L*_b_ is computed for the reference and target masks separately and their sum is added to the total loss. A weight *δ* was assigned to the binarity loss with its value determined empirically using grid search to ensure convergence. The total loss *L* is defined as:
$$\begin{array}{ccc} L & = & (L_{p(\mathrm{PIB})}+L_{p(\mathrm{NAV})}+L_{p(\mathrm{FBB})}+L_{p(\mathrm{FBP})}+L_{p(\mathrm{FMM})})/5\\ & & +\;\delta (L_{b(\mathrm{Reference})}+L_{b(\mathrm{Target})}) \end{array}$$

The masks are initialized using the original Centiloid reference and target mask and smoothed using a 4 mm FWHM Gaussian kernel. At each iteration, the masks are updated using the gradient information and smoothed again using the same 4 mm FWHM Gaussian kernel to ensure spatial consistency and remove spurious isolated voxels which are more likely to overfit the dataset. To ensure symmetry, both masks were averaged with their left‐right flipped version at each iteration. This was followed by a sigmoid function to clamp the masks to the [0–1] range. The optimization leverages pytorch's autograd engine for automatic computation of the gradients. Losses weights were initially set to *δ = *5e−4 and increased by a factor of 100 after 20 epochs, once the model started to converge. Using an initial smaller weight for the binary loss is necessary, as increasing its weight too early prevents the model from leaving its initial state. The random smoothing augmentation used to train DeepSUVR was also employed to reduce the variability due to different scanner's PSF. To facilitate convergence, the optimization is run using a 3‐level multiresolution approach, where the masks are first optimized using ×4 downsampled images. The masks are then upsampled and the optimization resumed with ×2 downsampled images, before finally being optimized on the full resolution images. For each resolution level, the optimization is stopped once the improvement in *L_p_* is less than 10e‐8 and the model has trained for a minimum of 1000 iterations at level 1, 3000 at level 2, and 8000 at level 3 (those were defined based on training without data augmentation, where the loss function is smooth and allowed to fully converge). Given that the optimization is only based on maximizing correlation with the corrected SUVR from DeepSUVR with no consideration for potential bias or scaling differences, there is no guarantee that the standard Centiloid transforms can be directly used. Instead, new Centiloids transforms need to be computed. To this end, the new SUVRs derived from these masks are recalibrated into Corrected SUVR using a linear regression against the DeepSUVR‐corrected SUVRs for each tracer, before being transformed into Centiloids using the standard transforms.

### 2.10 Evaluation

#### 2.10.1 Cross‐sectional analysis

We first evaluated the correlation between the Centiloids values derived from DeepSUVR (*CL*_DS_) and those from the Standard method (*CL*_Std_) for each tracer and in both the training and testing datasets using the coefficient of determination (*R* ^2^).

The correlation between PIB Centiloid values and those from each of the ^18^F tracers within the GAAIN calibration dataset was assessed using R^2^. A similar correlation analysis was performed for PIB and FBP in the OASIS dataset, specifically using matched scan pairs. A separate evaluation of the correlation analysis in the Aβ‐negative (PIB *CL*_std_ < 20CL) and Aβ‐positive (PIB *CL*_std_ ≥ 20CL) was conducted.

To assess the variability of the Aβ‐negative across tracers and studies, we fitted a 2‐Gaussian mixture model to each Centiloid distribution. The average of the mean and standard deviation of the first peak across all studies/tracers was then calculated.

To evaluate the stability of each method in estimating the mean and standard deviation of the first peak across studies and tracers, we performed a paired bootstrap analysis (*N* = 10,000). For each bootstrap iteration, we computed the standard deviation of the peak means and peak standard deviations across studies or tracers for each method. We then calculated the paired difference between each method and the reference method (*CL*_Std_) for each bootstrap. The distribution of these paired differences was used to estimate the average difference in stability and a nonparametric *p*‐value (based on the proportion of bootstrap samples with a sign opposite to the mean difference). A method was considered significantly more stable than *CL*_Std_ if the 95% confidence interval did not include zero, corresponding to *p *< 0.05.

The correlation between baseline Centiloid values and Mini‐Mental State Examination (MMSE) was evaluated using the Spearman rank correlation. We measured the effect size (Cohen's *d*) for differences in Centiloid values between Clinical Depression Rating (CDR) scale categories: 0 vs. 0.5, 0.5 vs. 1, and 0 vs. 1. The agreement between Centiloid values and visual reads in a subset of ADNI, ADNI‐DOD, A4, HABS‐HD, and AMYPAD were quantified using the area under the receiver operating characteristic curve (AUC). The corresponding CL thresholds that maximize the F1‐score were also computed. The F1‐score was selected to account for potential class imbalance. Furthermore, we used Cohen's *d* to measure the effect size of Centiloid differences between CERAD neuritic plaque score categories of “none/sparse” and “moderate/frequent”.

To assess whether any method provided a significant improvement over CL_Std_ for each of the above‐mentioned comparisons (correlation with MMSE, effect size between CDR levels, AUC and thresholds with visual reads, and effect size with CERAD^47^ categories), a paired bootstrap (*N* = 10,000) was conducted to generate non‐parametric *p*‐values.

### 2.11 Longitudinal trajectories

For studies with longitudinal data, we identified the number of longitudinal outliers based on the distribution of annual amyloid accumulation rates. The threshold was set at the 90^th^ percentile of the rate of change observed in AIBL study participants who underwent PIB PET scans exclusively on the same scanner (*N* = 168; AD = 20, CU = 92; MCI = 55). This approach established a reference range for Centiloid/year of [−5.8, 11.2]. Outliers were defined as consecutive visits of the same participant showing a decrease larger than 5.8CL/Year or an increase larger than 11.2CL/Year. The Hilbert‐Schmidt Independence Criterion (HSIC) and Spearman *ρ* were used to assess the correlation between the mean Centiloid and the rate of change between consecutive pairs of visits acquired at least 3 months apart. While Spearman *ρ* is nonoptimal to capture nonmonotonic relationships, it is more commonly used for assessing non‐linear associations and is easier to interpret. Therefore, both metrics are reported.

Within the A4 study, the effect size of the difference in amyloid accumulation rates between the treatment and placebo arms between the 2 month and the 56 month visits was measured using Cohen's *d*.

To assess whether any method provided a significant improvement over *CL*_Std_ for each of the above‐mentioned comparisons (HSIC and Spearman *ρ* between mean Centiloid and rate of change, Cohen's *d* between rate of CL accumulation in A4 treatment and placebo arms), a paired bootstrap (*N* = 10,000) was conducted to generate non‐parametric *p*‐values.

### 2.12 Detecting emerging Aβ pathology

This analysis aimed to quantify the proportion of participants with a baseline Aβ‐negative scan who subsequently progressed to Aβ‐positive status. Using a conservative *CL* threshold of 30 to define positivity,^48^, ^49^ participants with at least 4.5 years of follow‐up and a baseline Aβ‐negative scan (*CL* < 30), were classified as “Emergent Aβ+” if at least one follow‐up scan was Aβ‐positive (*CL*≥30); otherwise, they were classified as “stable Aβ‐negative”. The proportion of emergent Aβ‐positive participants, with a baseline Centiloid within each of the five CL brackets between 5 and 30CLs was reported.

### 2.13 Evaluating the new masks

To evaluate our approach, we first compared the DeepSUVR results to the Standard Centiloid in the test set of the AIBL–ADNI cross‐validation dataset. Those results informed the construction of new target and reference masks. CL computed using the new DeepSUVR masks (*CL*_DS mask_) were then calculated in the ten independent validation studies. These *CL*_DS Mask_ values were then compared to the Standard Centiloid values that used the WCb as the reference region (*CL*_Std_). Additional comparisons, detailed in the Supporting Information were conducted against Standard Centiloid values using the Composite WM+WCb reference region for FBP (*CL*_Comp_) and against our previously developed data‐driven approach based on the Nonnegative Matrix Factorization (*CL*_NMF_).

### 2.14 Assessing the risk of overfitting the data to the curve

A critical component of our model is the loss function *L*_c_, which constrains pairs of Centiloid values to align with an expected rate of change curve. This constraint introduces a potential risk of over‐correcting the Centiloid values thereby masking actual inter‐participant variability in amyloid accumulation trajectories. Although most of the existing literature has converged towards a unique trajectory, it is plausible that natural variations could be hidden by the noise and variability in the existing Aβ quantification methods. More advanced quantification techniques, such as DeepSUVR, might potentially allow their identification. To assess if DeepSUVR would over‐correct the Centiloid quantification in this scenario, we simulated a different trajectory in a subset of the population and retrained the model on the simulated data. To this end, each fold of the training dataset was randomly split in 2 subsets (20/80), with 20% of participants assigned a simulated slower or faster rate of change by a factor of 0.9, 1.1, 1.2, 1.5, and 2 by artificially scaling the number of days between each scans (subset 2), while the remaining participant kept their original rate of change (subset 1). New models were trained using each of the simulated dataset. The difference in the trajectory between the two populations was then assessed in the out‐of‐folds simulated AIBL+ADNI, using both the CL_Std_, and the newly trained DeepSUVR model to check that the difference in the simulated rate of accumulation was preserved. To further verify that the simulated trajectory was not learnt by the model, the trajectory of each DeepSUVR model inference on the real dataset, with no simulated accelerated trajectory was plotted. It is expected that in the real dataset, the trajectory between each subset remains the same. A 5^th^ order polynomial was fitted to each subset of participants to evaluate their trajectories and the peak of each polynomial used as a surrogate marker of the peak rate of accumulation, with bootstrapping used to assess confidence intervals.

## 3 RESULTS

### 3.1 Datasets

The demographics characteristics of the training (AIBL, ADNI) and independent testing cohorts are detailed in Table S1. The dataset for model development was derived from the combined AIBL and ADNI cohorts, including a total of 9266 amyloid PET scans from 3994 unique participants (3909 FBP, 1747 PIB, 902 FBB, 539 FMM, and 2169 NAV). For the model training set, we selected a subset of 7149 longitudinal PET scans from 2129 of these AIBL/ADNI participants with two or more PET visits, with scans acquired prior to November 2022 (3204 FBP, 1607 PIB, 451 FBB, 491 FMM, and 1396 NAV). Data from the remaining 1866 AIBL/ADNI participants, who only had a single PET imaging timepoint, or had multiple visits but were acquired after November 2022, were allocated to the internal test set, which was kept separate and not combined with the independent testing cohorts, but was combined with the training cohort cross‐validation results for all cross‐sectional and longitudinal analyses. The independent testing dataset included 15,807 PET scans (3957 FBP, 5201 PIB, 4926 FBB, 1275 FMM, and 448 NAV) from 10,543 unique participants from 10 external cohorts. The demographics of the training and testing sets are detailed in Table S2, and the demographic of each of the 5 sets used in the training and cross‐validation are summarized in Table S3.

### 3.2 Cross‐sectional analysis

The Centiloids obtained using DeepSUVR (*CL*_DS_) were strongly correlated with the Centiloids obtained from the standard method (CL_Std_) for all tracers in both the training cohort (*R* ^2 ^> 0.91) and the testing cohorts (*R* ^2 ^> 0.85) (Figure S4). The correlation was strongest for NAV, followed by PIB, FBB, FMM, and was weakest for FBP. We do however note a 10% under‐estimation of the Centiloid when using DeepSUVR compared to the standard method with NAV. The Bland‐Altman plot (Figure S5) revealed a small non‐linearity in the DeepSUVR correction compared to the standard method, with a small increase at around 50CL and a small decrease above 100CL, which is more obvious in NAV population given its higher proportion of high CL scans, and seems to be driving the above‐mentioned 10% under‐estimation in NAV. It should also be noted that 91 out of 546 participants imaged using NAV switched from a Philips Gemini scanner to a Siemens Vision scanner. Our previous study showed that the Vision scanner leads to a 13% increase in CL compared to Gemini[7]. This would similarly increase the rate of change when the switch happened, which could also lead to the scaling down the NAV CL values to bring them closer to the curve, potentially biasing the Centiloid estimate.

### 3.3 Head‐to‐head tracer comparison

**FIGURE 3:**
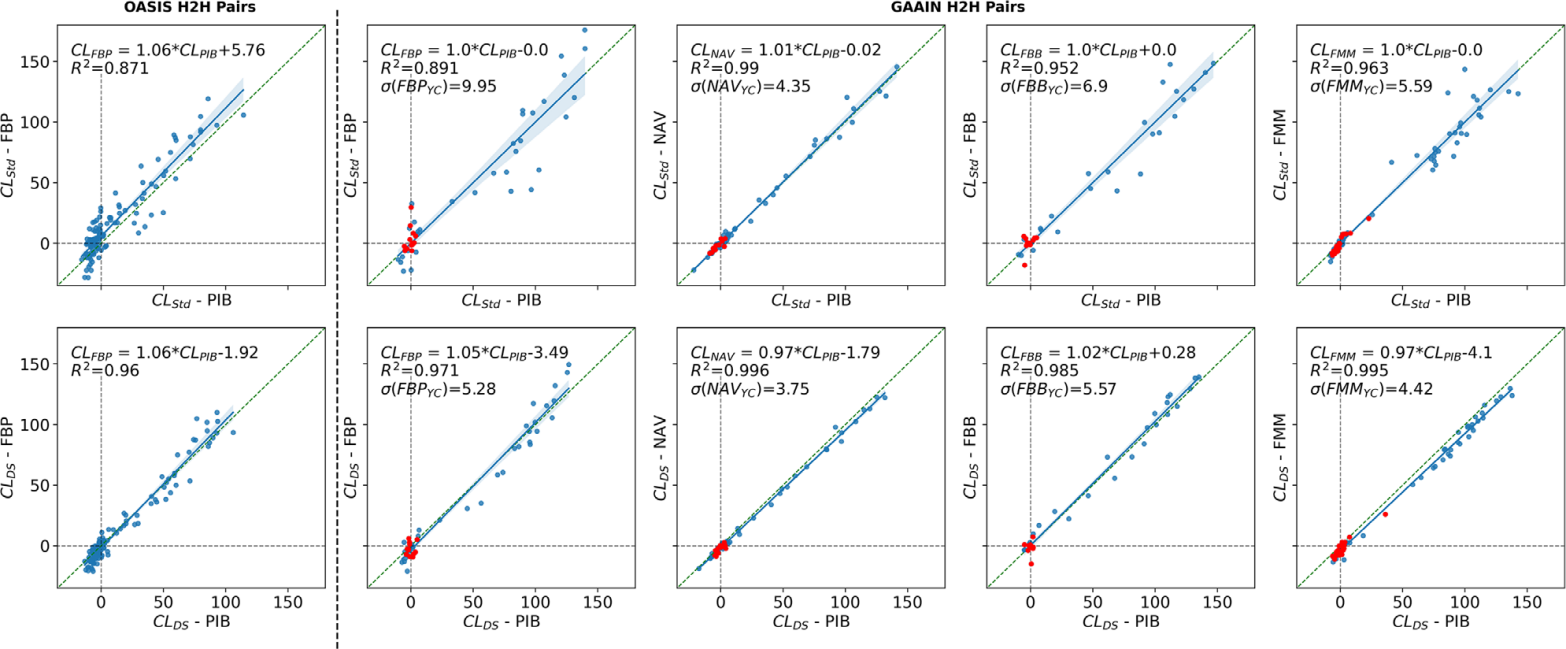
Scatterplots comparing PIB vs. FBP in the OASIS Head‐to‐Head (H2H) dataset (left) and each ^18^F‐Tracer vs. PIB in the GAAIN Head‐to‐Head Calibration dataset (Right), using CL_Std_ (top) and CL_DS_ (bottom). The correlation between each pair of tracers is assessed using the coefficient of determination *R* ^2^. The standard deviation in the young controls (YC) for each tracer is denoted as σ(Tracer_YC_). Note that for FMM, one YC with a CL > 20 on both PIB and FMM across all methods was determined to be an outlier and excluded from the standard deviation calculation. FBP, ^18^F‐Florbetapir; FMM, ^18^F‐Flutemetamol; OASIS, the Open Access Series of Imaging Studies; PIB, ^11^C‐PiB.

Using data from 120 OASIS study participants with repeat PIB and FBP scans acquired within 7 months of each other, and from 289 GAAIN Calibration dataset participants scanned with PIB and one of the ^18^F tracers, the scatterplot in Figure 3 illustrates the agreement between each ^18^F tracer and PIB. *CL*_DS_ demonstrated the highest correlation between each ^18^F tracer and PIB; within the GAAIN Calibration dataset, all tracers achieved an *R* ^2^ above 0.97 with PIB. Compared to the standard method, the greatest improvement in the coefficient of determination was observed with FBP (from *R* ^2 ^= 0.89 to *R* ^2 ^= 0.97), followed by FBB (from *R* ^2 ^= 0.95 to *R* ^2 ^= 0.98) and FMM (*R* ^2 ^= 0.96 to *R* ^2 ^= 0.99) and with a marginal improvement for NAV (*R* ^2 ^= 0.990 to *R* ^2 ^= 0.996). A similar improvement in *R* ^2^ between FBP and PIB was observed in the OASIS head‐to‐head dataset, increasing from 0.87 to 0.96. None of the other evaluated methods achieved higher agreement between any pair of tracers (Figure S6). Furthermore, the standard deviation in the young controls was smallest using DeepSUVR for all tracers, except for FBB, where the NMF method achieved the lowest standard deviation. Bland Altman plot (Figure S7) revealed a negative bias of −5.35CL with DeepSUVR in FMM, which likely reflects a scanner bias. The GAAIN calibration scans for NAV, FBB and a subset of FBP were acquired on AIBL scanners, whereas FMM scans were acquired on a completely different set of scanners, and therefore different from the ones used to acquire the FMM in AIBL used to train the model. It is therefore likely that DeepSUVR “corrected” their quantification to better align with the other AIBL scans.

In the sub‐group analysis in the Aβ‐negative (Figure S8), *CL*_DS_ showed stronger correlation than *CL*_Std_ between PIB and each tracer, except for FMM where it was considerably lower (*R* ^2 ^= 0.63** vs**. *R* ^2 ^= 0.87), although the slope was closer to unity with *CL*_DS_ compared to *CL*_Std_ (1.08 vs. 1.42). A similar strong different in slope was seen in the FBP vs. PIB, with slopes of 1.4 and 1.64 in OASIS and GAAIN using *CL*_Std_ *c*ompared to 1.08 and 1.21 using *CL*_DS_ *_._*

In the Amyloid positive (Figure S9), *CL*_DS_ showed stronger correlation between each pair of tracers compared to *CL*_Std_ and every other quantification method.

### 3.4 Agreement with visual reads

The area under the curve (AUC) values, reflecting agreement between visual PET reads (from ADNI, ADNI‐DOD, A4, HABS‐HD, and AMYPAD) and the positivity based on Centiloid values obtained using each quantification method are presented in Table 1. Although the AUC values were high for all quantification methods, *CL*_DS_ consistently yielded the highest AUC across all cohorts, with significant improvement over *CL*_Std_ in A4, ADNIDOD, HABS‐HD, and AMYPAD FMM. The corresponding thresholds that maximize the F1‐scores are presented in Table S4. It shows that for each study, similar thresholds were obtained across methods, with no significant differences in the thresholds obtained between CL_DS_ and CL_Std_.

**TABLE 1:** AUC values for agreement between visual PET reads and the Centiloid values from different quantification method across specified cohorts.

**Parameter**	**A4**	**ADNI**	**ADNIDOD**	**HABS‐HD**	**AMYPAD**	**AMYPAD**
*CL*_Std_	0.919	0.973	0.915	0.989	0.962	0.974
*CL*_Comp_	0.951^***^	0.965	0.927	–	–	–
*CL*_NMF_	0.948^***^	0.972	0.928	0.993^***^	0.975^***^	0.974
*CL*_DS_	**0.954** ^***^	**0.978**	**0.942** ^*^	**0.994** ^***^	**0.982** ^***^	**0.979**
Tracers	FBP	FBP/FBB	FBP	FBB	FMM	FBB
N	1772	80/58	230	1094	1264	994
Visual Positivity (%)	35.0	48.6	30.9	7.8	24.0	19.3

Abbreviations: ADNI, Alzheimer's Disease Neuroimaging Initiative; ADNI‐DOD, the Department of Defense Alzheimer's Disease Neuroimaging Initiative; AMYPAD, Amyloid Imaging to Prevent Alzheimer's Disease Consortium; HABS‐HD, the Health and Aging Brain Study: Health Disparities.

*Note*: The highest AUC for each cohort is highlighted in bold. The statistical significance over *CL*_Std_ based on bootstrapping is indicated using:

* *p* < 0.05.

** *p* < 0.01.

*** *p* < 0.001 (^18^F‐Florbetapir (FBP) and ^18^F‐Florbetaben (FBB) were grouped together for ADNI due to the otherwise small numbers for statistical analysis). CL_Comp_ is only reported for cohorts using FBP.

### 3.5 Agreement with neuropathology

The effect size (ES; Cohen's d) between FBP CLs from ADNI participants (N = 53), stratified by CERAD diffuse plaques scores (‘none/sparse’ (*N* = 9) vs. ‘moderate/frequent’ (*N* = 44)), are presented for each quantification method in Figure S10. While the ES were high for all quantification methods, the highest ES was obtained using *CL*_DS_, and was significantly higher than the one obtained using C*L*_Std_ (Cohen's *d* = 3.70 vs. 2.79, *p* < 0.001).

### 3.6 Baseline Centiloid distribution

**FIGURE 4:**
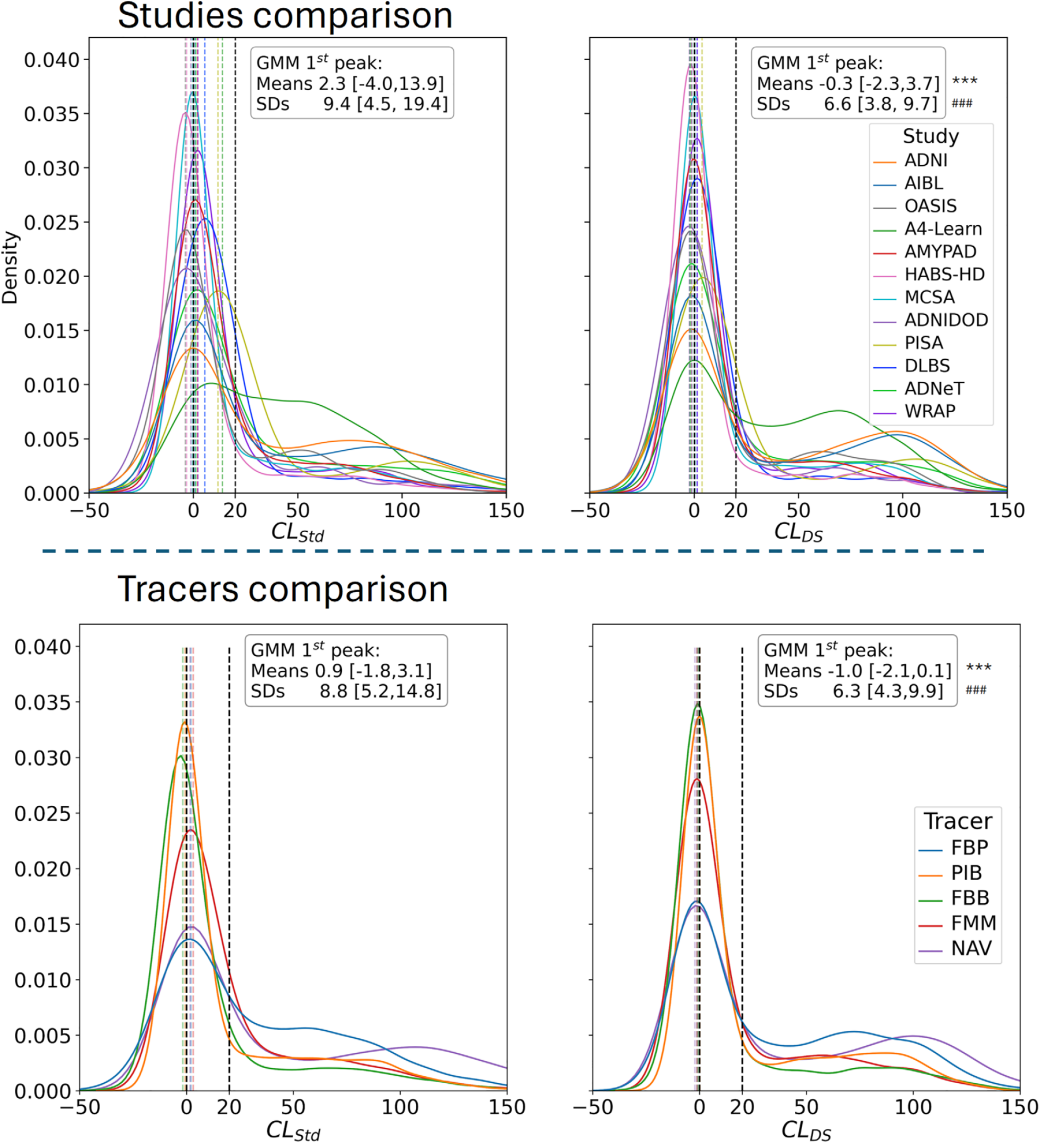
Histogram distribution of baseline Centiloid values across the 12 cohorts using the Standard and DeepSUVR CL methods. For each approach, a Gaussian mixture is fitted to the distribution of Centiloid values of each study (top) and each tracer (bottom), and the average mean [min, max] and average standard deviation [min, max] of the first peak across all studies (tracer respectively) is reported. The dashed lines mark the 0CL and 20CL. Significantly lower variability in the means and standard deviations when comparing CL_DS_ to CL_Std_ across studies/tracers based on bootstrapping are indicated using: *** (*p* < 0.001) for lower variabilities in the means and ^###^ (*p* < 0.001) for lower variabilities in the standard deviations.

The baseline distribution of tracers per study was as follow: A4‐Learn (FBP = 12.3%); ADNI (FBB = 2.5%, FBP = 9.1%, IB = 0.6%); ADNIDOD (FBP = 1.6%); ADNeT (NAV = 3.0%); AIBL (FBB = 1.0%, FBP = 3.3%, FMM = 1.7%, NAV = 5.6%, PIB = 3.6%); AMYPAD (FBB = 4.0%, FMM = 5.4%); DLBS (FBP = 2.0%); HABS‐HD (FBB = 21.2%); MCSA (PIB = 12.4%); OASIS (FBP = 1.3%, PIB = 4.1%); PISA (FBB = 1.7%); WRAP (NAV = 0.0%, PIB = 3.6%). The distributions of the baseline Centiloid values computed using the Standard method and DeepSUVR across all studies and tracers are presented in Figure 4. To assess the variability of the Aβ‐negative across studies, a two‐Gaussian mixture model was fitted to each distribution. The average mean and standard deviation of the first peak (used as a proxy of the Aβ‐negative Centiloid distribution), averaged across all studies, are reported in Figure 4. CL_DS_ showed significantly more consistent means for the first peak, with all peaks contained within a 6 CL unit range from [−2.3CL to 3.7CL] across studies, compared to a 15 CL unit range when using *CL*_Std_ from [−4.0CL to 11.6CL]. Similarly, *CL*_DS_ showed a significantly smaller standard deviation in the Aβ‐negative peak with an average standard deviation of 6.5 (range of [3.8–9.7]) compared to 9.0 (range of [4.5–14.5]) for *CL*_Std_. The distributions computed using the other quantification approaches are presented in Figures S11 and S12, showing that using the *CL*_Comp_ and *CL*_NMF_ both led to some reduction in variability in the Aβ‐negative compared to *CL*_Std_, it was still much higher than with *CL*_DS_.

### 3.7 Correlation with cognitive measures

Spearman rank correlations between baseline Centiloid values and MMSE scores in the external testing cohorts are reported in Table S**5**. The strongest association between Centiloid and MMSE was obtained using *CL*_DS_ (ρ = −0.111) which was significantly stronger than CL_Std_ (ρ** = **‐0.077, *p *< 0.001). Similarly, the largest effect sizes (Cohen's *d*) between CDR 0 vs 0.5, CDR 0.5 vs. 1 and CDR 0 vs 1 were all achieved using CL_DS_ (0.303, 0.718, and 1.061, respectively), which were all significantly higher than CL_Std_ (0.272, 0.644, and 0.960, respectively, all with *p *< 0.001). Similar findings were obtained in the training cohorts (Table S6). While the associations between CL and cognition are not directly comparable between the training and testing dataset due to different recruitment criteria, both the training and testing sets consistently show stronger associations between CL and cognition when using DeepSUVR.

### 3.8 Longitudinal trajectories

**FIGURE 5:**
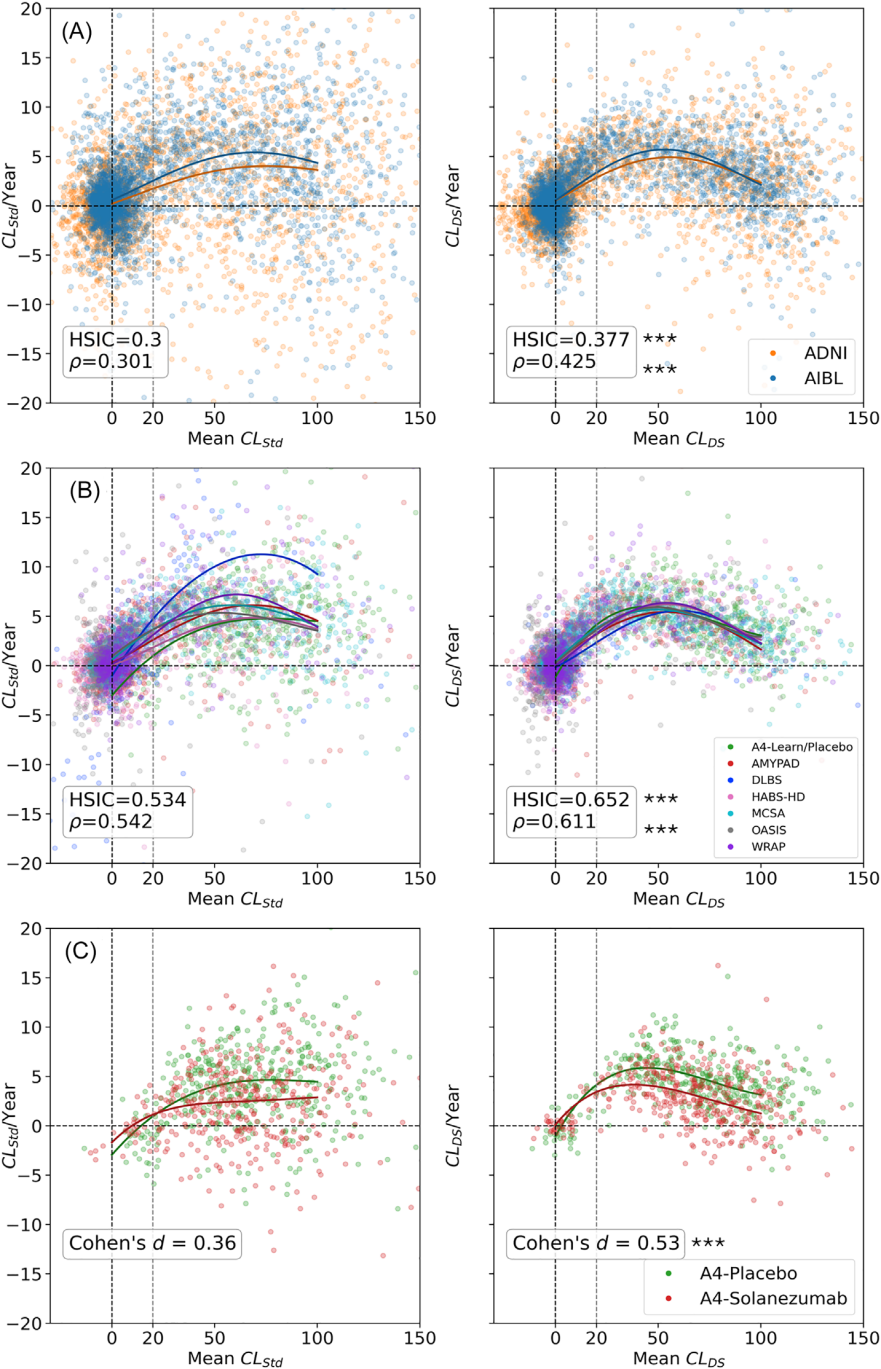
(a) Rate of CL change per year compared to mean CL computed from the 2 training (ADNI (Subjects = 48.6% FBB = 5.5%, FBP = 36.4%, PIB = 2.7%); AIBL (Subjects = 51.4% FBB = 1.9%, FBP = 8.9%, FMM = 7.9%, NAV = 13.3%, PIB = 23.4%)) cohorts, (b) the 7 testing cohorts with longitudinal data (A4‐Placebo (Subjects = 13.4% FBP = 10.3%); AMYPAD (Subjects = 22.1% FBB = 9.1%, FMM = 10.5%); DLBS (Subjects = 5.0% FBP = 5.4%); HABS‐HD (Subjects = 17.2% FBB = 13.2%); MCSA (Subjects = 23.4% PIB = 25.9%); OASIS (Subjects = 10.0% FBP = 1.5%, PIB = 11.1%); WRAP (Subjects = 8.9% PIB = 13.2%)), (c) and the placebo and treatment arms of the A4 Study: A4‐Placebo (Subjects = 51.7% FBP = 51.7%); A4‐Solanezumab (Subjects = 48.3% FBP = 48.3%) using the Standard (left) and DeepSUVR CL (right) methods. Each point represents the mean and rate of change between a pair of consecutive visits from the same participant. Each curve shows a 5th order polynomial fitted to each study. The Hilbert‐Schmidt Independence Criterion between Mean CL and CL/Year is denoted using HSIC, while the Spearman rank correlation is denoted using r (a and b), while the effect size of the CL accumulation per year between the 2 arms of the A4 study is denoted using Cohen's d (c). The vertical dashed lines mark the 0CL and 20CL, while the horizontal dashed line mark the 0CL/Year. Significantly higher correlation or effect size when comparing *CL*_DS_ to *CL*_Std_ across studies/tracers based on bootstrapping are indicated using: *** *p* < 0.001. AMYPAD, the Amyloid imaging to prevent Alzheimer's disease; DLBS, the Dallas Lifespan Brain Study; FBB, ^18^F‐Florbetaben; FBP, ^18^F‐Florbetapir; FMM, ^18^F‐Flutemetamol; HABS‐HD, the Health and Aging Brain Study: Health Disparities; HSIC, Hilbert‐Schmidt Independence Criterion; MCSA, the Mayo Clinic Study of Aging; NAV, ^18^F‐NAV4694; PIB, ^11^C‐PiB; WRAP, the Wisconsin Registry for Alzheimer's Prevention.

Rate of annual CL change (CL/Year) vs. mean *CL* values for pairs of consecutive visits, using the standard and DeepSUVR CL quantifications, for the training and testing cohorts are presented in Figure 5a and Figure 5b, respectively. A comparison across all quantification methods is provided in Figures S13 and S14. The HSIC, which is used assess nonlinear statistical dependence between the mean CL and CL/Year was the highest for DeepSUVR in both training (HSIC = 0.377) and testing cohorts (HSIC = 0.651) and were significantly higher than those from CL_Std_ (HSIC = 0.300 in training, HSIC = 0.532 in testing, *p *< 0.001), with similar findings using Spearman rank correlation *ρ*. To visually assess the concordance between the participants’ longitudinal trajectories in each study, a 5^th^ order polynomial was fitted to each study, and the resulting trajectory plotted in Figure 5. Those curves further emphasise that the trajectories derived from each cohort are more concordant when using DeepSUVR. Additionally, the distribution of annual *CL_Std_* change from consecutive AIBL PIB scans acquired on the same scanner was used to establish a 95% confidence interval for expected annual changes, accounting for measurement noise, when no change of scanner or tracer occurs. The resulting threshold of –5.8CL/Y and 11.2CL/Y were used to assess the percentage of longitudinal outliers in both the training and testing datasets and are presented in Table 2, showing reduction from three‐ to five‐fold in the percentage of outliers when using DeepSUVR compared to the Standard CL in both the training and testing cohorts. Spaghetti plots, with negative outlier trajectories marked in red and positive outlier trajectories marked in blue, are presented in Figure S15 for the training set and Figure S16 for the testing set.

**TABLE 2:** Percentage of outliers based on the 95% confidence interval of yearly change in CL_Std_ measured in ^11^C‐PiB (PIB) in the Australian imaging, biomarkers, and lifestyle study of ageing (AIBL), when no change of tracer or scanner occurred.

	**Training cohort**		**Testing cohort**	
**Parameter**	Neg outliers (%)	Pos outliers (%)	Neg outliers (%)	Pos outliers (%)
*CL_Std_*	6.45	6.74	2.48	1.76
*CL_Comp_*	5.84	4.95	1.80	1.26
*CL_NMF_*	4.70	3.69	1.17	1.22
*CL_DS_*	**1.58**	**1.92**	**0.48**	**0.50**

### 3.9 A4 study

Longitudinal trajectories for the treatment and placebo arms of the A4 study, between the 2‐month and 56‐month visits, using the standard and DeepSUVR CL quantifications are presented in Figure 5c, along with the effect size for the difference in annual CL accumulation between the two arms. A comparison across all quantification methods is shown in Figure S17, and a violin‐plot of the rate of change in Figure S18. The largest effect size was obtained using DeepSUVR (Cohen's *d* = 0.53), which was significantly higher than the one obtained using *CL*_Std_ (Cohen's *d* = 0.36, *p *< 0.001). Notably, participants with an Aβ‐negative baseline scan, defined as *CL* < 20 using each method's CL value, exhibited significantly less variability (lower standard deviation) in their rate of change using DeepSUVR (SD(*CL_DS_*/Year) = 4.96 compared to SD(*CL*_Std_/Year) = 7.77, *p *< 0.05).

### 3.10 Detecting emerging Aβ pathology

Using five *CL* brackets between 5 and 30CLs, the proportion of emergent Aβ‐positive participants with a baseline CL within each bracket is presented in Table S7 for the testing set and Table S8 for the training set. All participants with a baseline CL_DS_ between 20 and 30 were classified as emergent Aβ‐positive in training set and 98% in the testing set. In contrast, using CL_Std_, only 85% of the participants in the Training set and 69% in the Testing set were emergent Aβ‐positive, with the remaining classified as stable Aβ‐negative. In the 15‐20CL bracket, 81% and 91% were emergent Aβ‐positive using *CL*_DS_ in training and testing cohorts respectively, compared to 48% and 69%, respectively using CL_Std_. The trajectories of the emerging Aβ‐positive and stable Aβ‐negative for all methods are presented in Figure S19 for the testing cohorts and Figure S20 for the training cohorts, further illustrating the reduced variability in the *CL*_DS_ longitudinal trajectories.

### 3.11 Simulation study

The results of the simulation study are presented in Figure S21. When the models were evaluated on the out‐of‐folds simulated data, the simulated rate of change was mostly preserved with a measured difference in peak accumulation of 0.85, 1.09, 1.20, 1.47, and 1.81 (compared to the introduced rate of 0.9, 1.1, 1.2, 1.5, and 2). When those models were evaluated on the real data, where a single trajectory is expected, the derived trajectories had peaks within 10% of each other's, and aside from the most extreme case (×2), within each other's confidence interval.

### 3.12 DeepSUVR derived masks

**FIGURE 6:**
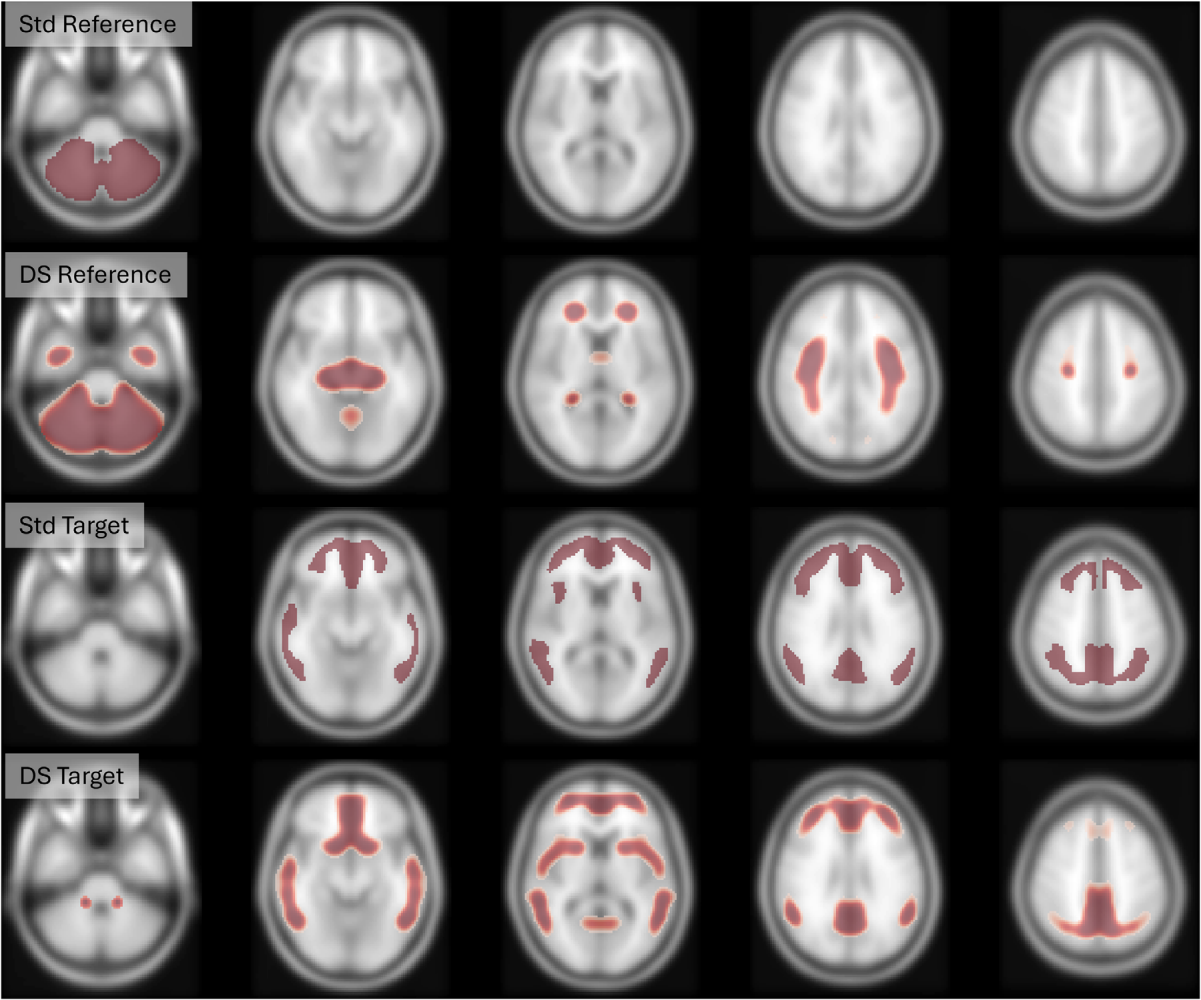
Axial views of the Standard CL reference mask (row1) and the new reference mask derived from DeepSUVR (row2), the Standard CL target mask (row3), and the new target mask derived from DeepSUVR (row4).

The novel reference and target masks optimized to maximize their correlation with the DeepSUVR‐derived SUVRs from AIBL+ADNI dataset across all five tracers are presented in axial views in Figure 6, and coronal and sagittal views in Figures S22 and S23, respectively. The DeepSUVR‐derived target mask showed a high degree of overlap with the standard Centiloid neocortical mask (Dice coefficient = 0.67). The new target mask showed less spread in some regions of the frontal and parietal lobes, but larger coverage in the orbitofrontal and precuneus, areas known for very early amyloid accumulation. There was also larger coverage in the temporal lobe and the deep grey matter structures including the caudate and putamen. In contrast, the derived reference mask, while including the whole cerebellum, also included extensive regions of the subcortical white matter, including part of the anterior and superior corona radiata tracts, and a small region in temporal pole white matter, resulting in less overlap with the standard reference mask (Dice coefficient = 0.50). It also included the ventral diencephalon but did not extend to the brain stem.

The Centiloids values derived using these new masks (CL_DS mask_) showed a very high correlation with CL_DS_ across all tracers, ranging from *R* ^2 ^= 0.987 to 0.995 in the training dataset and ranging from *R* ^2 ^= 0.983 to 0.997 in the testing datasets with no bias (Scatter and Bland‐Altman plots in Figure S24 and S25). The Centiloid transforms used to convert the SUVR_DS mask_ into *CL*_DS mask_ are listed in Table S9.

Although these new masks did not exactly replicate the performance of direct DeepSUVR quantification, they generally performed similarly to DeepSUVR and significantly outperformed *CL*_Std_ in all evaluations where *CL*_DS_ significantly outperformed *CL*_Std_. Specifically, in the GAAIN Calibration dataset, *CL*_DS mask_ showed very high correlation between each ^18^F tracer and PIB, with all tracers showing a *R* ^2^ above 0.95, and a low standard deviation in the young controls (< 6.2) (Figure S**6**). It also yielded the second highest AUC for agreement with visual reads across all studies (Table S10) and the second highest effect size for discriminating CERAD none‐sparse and moderate‐frequent stages (Figure S10). For baseline Centiloid distribution, *CL*_DS mask_ had the second lowest average standard deviation across studies (*CL*_DS mask_ 7.7 vs *CL*_DS_ 6.5) and across tracers (*CL*_DS mask_ 7.4 vs *CL*_DS_ 6.3) (Figures S11 and S12 respectively). It also achieved the second highest correlation with MMSE (*CL*_DS mask_ ρ = −0.093 vs. *CL*_DS_ ρ = −0.111), the second largest effect size between CDR 0 and 0.5 (*CL*_DS mask_ 0.285 vs. *CL*_DS_ 0.303), the largest effect size between CDR 0.5 and 1.0 (*CL*_DS mask_ 0.727 vs. *CL*_DS_ 0.718), and second largest effect size (ES) between CDR 0 and 1.0 (*CL*_DS mask_ ES = 1.055 vs. *CL*_DS_ ES = 1.061) (Table S5).

When used for longitudinal analysis, *CL*_DS mask_ had the third highest HSIC in the training (*CL*_DS mask_ HSIC = 0.375 vs. *CL*_DS_ HSIC = 0.377) and second highest in the testing cohorts (*CL*_DS mask_ HSIC = 0.635 vs *CL*_DS_ HSIC = 0.651) (Figures S13 and S14 respectively). This was also associated with the second lowest percentage of longitudinal outliers (Table S11). In the A4 dataset, *CL*_DS mask_ led to the second highest effect size between the 2 arms of the study (*CL*_DS mask_ ES = 0.52 vs. ES = 0.53) (Figure S17).

## 4 DISCUSSION

In this work, we present DeepSUVR, a deep learning technique designed to learn a SUVR correction factor to improve the longitudinal consistency of Aβ PET measurements, thereby improving inter‐tracer agreement. This work also addresses a machine learning challenge of improving quantification in scenarios where definitive ground truth is unavailable, or where the available ground truth may be noisy or biased. The novelty of this approach lies in leveraging expected population‐level longitudinal amyloid trajectories to correct the measurements that constitute these trajectories. By limiting the use of the longitudinal information to the loss function during training, the resulting network does not require longitudinal data at inference time, significantly broadening its applicability and reducing the risk of over‐fitting to specific longitudinal trajectories. Though specifically applied here to Aβ PET, this approach might be of broad value for other aging related biomarkers where ground truth is not readily available but longitudinal trajectories are expected to be constrained.

Another significant contribution of this work is to address the black‐box nature of the deep learning algorithm used. By optimizing new reference and target masks that largely emulate DeepSUVR's corrected quantification, and demonstrating their use in quantification in external datasets, provides a tangible means to interpret the model's impact. While this is an indirect explainability, as it does not attempt to directly understand the model's decisions such as saliency or activation maps, it provides a practical and easy‐to‐understand approach to interpretability. Another benefit of the masks is that they could be more easily integrated into existing pipelines, while providing most of the performances of the full DeepSUVR network. The most striking difference between the DeepSUVR‐derived masks and the standard CL mask is the inclusion of subcortical WM in the reference mask. While it is well documented that the Composite WM can improve FBP quantification,^6^, ^13^, ^50^ it has also been shown to improve longitudinal quantification with PIB^51^ and FBB.^52^ This improvement is likely due to a combination of factors, including spill‐in contamination of the WM into the Cerebral GM that can be compensated by using the WM in the reference region,^53^ or potential differences in between‐scan head positioning, with the subcortical WM being closer to the center of the scanner field of view and therefore less affected by repositioning.^54^DeepSUVR demonstrated reduced intra‐ and inter‐study variability both cross‐sectionally (evidenced by lower variance in the Aβ‐negative peaks across studies and tracers) and longitudinally (indicated by increased HSIC and Spearman rank correlation of the longitudinal trajectories with fewer outliers). A beneficial consequence of this reduced variability was an increased inter‐tracer agreement in both the GAAIN and OASIS head‐to‐head datasets, as well as a reduction in the variance in the GAAIN young controls. These improvements collectively contributed to a narrower distribution of the Aβ‐negative group across all cohorts. The increasing reliance on Centiloid values for clinical diagnosis and treatment eligibility underscores the critical need for improved concordance among different tracers to ensure consistent decision‐making. An Aβ quantification that is more sensitive to longitudinal changes will also improve monitoring of therapeutic responses. With an increasingly large body of work trying to model the natural history of Aβ accumulation in relation to different covariates or other biomarkers, improving the concordance between studies as well as reducing their longitudinal variability also means that we will be able to better combine and harmonise large datasets, increasing the power to detect subtle effects on amyloid accumulation rates or age of onset.

The inherent non‐linearity and ‘black‐box’ nature of deep‐learning technique raise valid concerns on potential model overfitting and removal of biological variability in the participants’ trajectories. Such effects could obscure true differences between sub‐groups (such as based on *APOE* status, sex, age, ethnicity, etc…), and prove detrimental to understanding differences in response to therapy.^55^ Our simulation study showed that if a small proportion of the population within the training data exhibited a distinct rate of accumulation, the DeepSUVR model largely preserved this unique trajectory rather than forcing conformity with the majority pattern. These results indicate that DeepSUVR primarily mitigates variability stemming from noise, errors in spatial normalization or differences in tracers and scanner, rather than suppressing participants’ inherent biological variability. These findings were further supported by our analysis on the A4 study, where distinct trajectories between the placebo and treatment arms were observed, with DeepSUVR providing the largest difference in their respective rates of change.

Although the relationship between Aβ burden and cognition are typically considered indirect, the increased correlation with MMSE, and increased effect size between CDR 0, 0.5, and 1 provide further evidence that DeepSUVR may be providing a more accurate estimate of Aβ burden.

## 5 LIMITATIONS

While this study used two large datasets for training and 10 for testing, one of the main limitations is the imbalanced representation of PET tracers in the datasets. In particular, FBP was over‐represented in the training set (45%), while PIB, FBB, and FBP were most common in the testing set (33%, 31%, and 25%, respectively). While NAV was well represented in the training set (23%), its representation in the external datasets was limited (3%), and no external study had longitudinal NAV. DeepSUVR's performance in external NAV datasets therefore needs to be further explored.

The use of different PET scanners has also started to emerge as a potential factor affecting quantification.^7^, ^56^ In this work, we attempted to alleviate the impact of using different scanners by using random smoothing as part of the training scheme. While this can account for differences in resolution, it cannot account for differences due to other factors such as scatter correction and different reconstruction methods.^7^ As noted with the NAV results, this could impact the training of DeepSUVR, where changes in scanner can artificially increase or decrease the apparent rate of change, potentially leading to an over or under‐correction during the training of DeepSUVR to bring them closer to the curve. DeepSUVR could potentially be trained to use scanner information, but this is not trivial since 32 scanners were used in the training set alone, with different reconstruction parameters, and with only a handful present in the testing sets, raising the question of how to generalize the model to unobserved scanners. Scanners and tracers are also often well correlated, as sites tend to favor a single tracer per scanner, making it harder to disambiguate the scanner effect from the tracer effect. One potential approach could be to use a simplified grouping based on the scanner technology, focusing on factors that are most likely to be driving significant differences such as analogue vs. digital detector, PET‐CT vs. PET‐MR, or different type of reconstructions, such as Time‐of‐Flight (ToF) vs nonToF. This was however outside the scope of this current work.

Another limitation of this approach is the reliance on the SPM pipeline which can require manual intervention to fix incorrect spatial normalization. While training DeepSUVR using more robust preprocessing pipelines was outside the scope of this study, we do not foresee any issues with training such models which we will investigate in future work. It should however be noted that deriving DeepSUVR mask currently requires spatially normalized images. It would therefore not be applicable to methods that do not include this step, such as methods based on Freesurfer.

It is unfortunately impossible to categorically state that the DeepSUVR model only removes noise and does not remove any meaningful longitudinal variability, since this is what it was designed to do. It is however worth noting that the model improved concordance with visual reads, neuropathology and cognition, tasks that the model was neither trained nor designed to improve on. This indicates that if any meaningful variability is lost, the improvement in agreement with visual reads and neuropathology alone would be still beneficial in clinical settings and trials, compared to the standard approach.

Finally, we were unable to test the model on data from patients treated with monoclonal antibody. While we expect the DeepSUVR to work on those, future validation will need to be conducted when such data becomes available.

## 6 CONCLUSION

This work demonstrates that deep learning provides a significant improvement in PET quantification of Aβ burden, outperforming standard methods both cross‐sectionally and longitudinally in both observational and interventional studies. DeepSUVR enhances the harmonization of large datasets and different PET tracers, while also reducing longitudinal variability. This will allow better pooling of datasets and improve our ability to detect differences in trajectories between subgroups. A superior Centiloid harmonization will also allow researchers to better validate and compare existing and novel plasma and other biomarkers for Aβ. With the advent of disease modifying therapies, Aβ PET is gaining an increasingly important role for both qualifying patients for treatment and as an outcome measure. More robust and harmonized quantification will allow consistent decision making across centers. Additionally, the reduction in longitudinal variability will also be critical in trials where the outcome measure of an intervention is expected to be subtle such as altering the rate of accumulation.

## CONSENT STATEMENT

Participants in all 12 studies signed informed consent.

## CONFLICT OF INTEREST STATEMENT

Pierrick Bourgeat, Jurgen Fripp, Leo Lebrat, and Vincent Dore have filled a patent application based on the technology described in this manuscript (WO 2025/129253). Christopher Rowe has received research grants from Avid, Piramal Imaging, GE Healthcare, Cerveau, Enigma, Eisai, and Biogen. Victor Villemagne is and has been a consultant or paid speaker at sponsored conference sessions for Eli Lilly, Piramal Imaging, Life Molecular Imaging, GE Healthcare, Abbvie, Lundbeck, Shanghai Green Valley Pharmaceutical Co Ltd, IXICO, and Hoffmann La Roche. John C. Morris is funded by NIH grants # P30 AG066444; P01AG003991; P01AG026276; U19 AG032438; and U19 AG024904. Neither Dr. Morris nor his family owns stock or has equity interest (outside of mutual funds or other externally directed accounts) in any pharmaceutical or biotechnology company. Duygu Tosun has nothing to disclose relevant to this work. Sterling Johnson has consulted for Eli Lilly, AlzPath, and Enigma Biomedical. Christopher Schwarz receives research funding from the NIH. Gill Farrar and Ariane Bollack are full‐time employees of GE HealthCare. Frederik Barkhof is a member of the steering committee or Data Safety Monitoring Board for Biogen, Merck, Eisai and Prothena; Advisory board member for Combinostics, Scottish Brain Sciences, Alzheimer Europe; Consultant for Roche, Celltrion, Rewind Therapeutics, Merck, Bracco. Research agreements with ADDI, Merck, Biogen, GE Healthcare, Roche; Co‐founder and shareholder of Queen Square Analytics LTD.

The other authors declare no conflicts of interest. Author disclosures are available in the Supporting Information.

## Supporting information



Supporting Information

Supporting Information

## Data Availability

A subset of the AIBL data can be downloaded through LONI after registration at http://adni.loni.usc.edu/category/aibl‐study‐data/ ADNI, ADNI‐DOD, HABS‐HD, MCSA, WRAP data can be downloaded through LONI after registration at https://ida.loni.usc.edu/ OASIS3 data can be downloaded through NITRC after registration at https://sites.wustl.edu/oasisbrains/ AMYPAD data can be requested via an EOI on the Alzheimer's Disease Data Initiative (ADDI) Workbench https://amypad.eu/data/ A4 data can be requested after registration at a4studydata.org DLBS data can be downloaded on the OpenNeuro website https://openneuro.org/datasets/ds004856/versions/1.2.0 PISA data can be requested by contacting the investigators to submit an EOI, with sharing also requiring a data sharing agreement. The python code used to run the NMF models can be downloaded from https://doi.org/10.25919/5f8400a0b6a1e The DeepSUVR model and derived masks are free to use for non‐commercial use and can be downloaded from https://github.com/csiro/DeepSUVR
