# Supplementary material for: AI‐enhanced Centiloid quantification of amyloid PET images
Source: Alzheimers Dement. 2026 Feb 11;22(2):e71162. doi: 10.1002/alz.71162 (PMC12892302; doi:10.1002/alz.71162)
Supplement: Supplementary file 1 — Supporting Information [file ALZ-22-e71162-s002.docx]

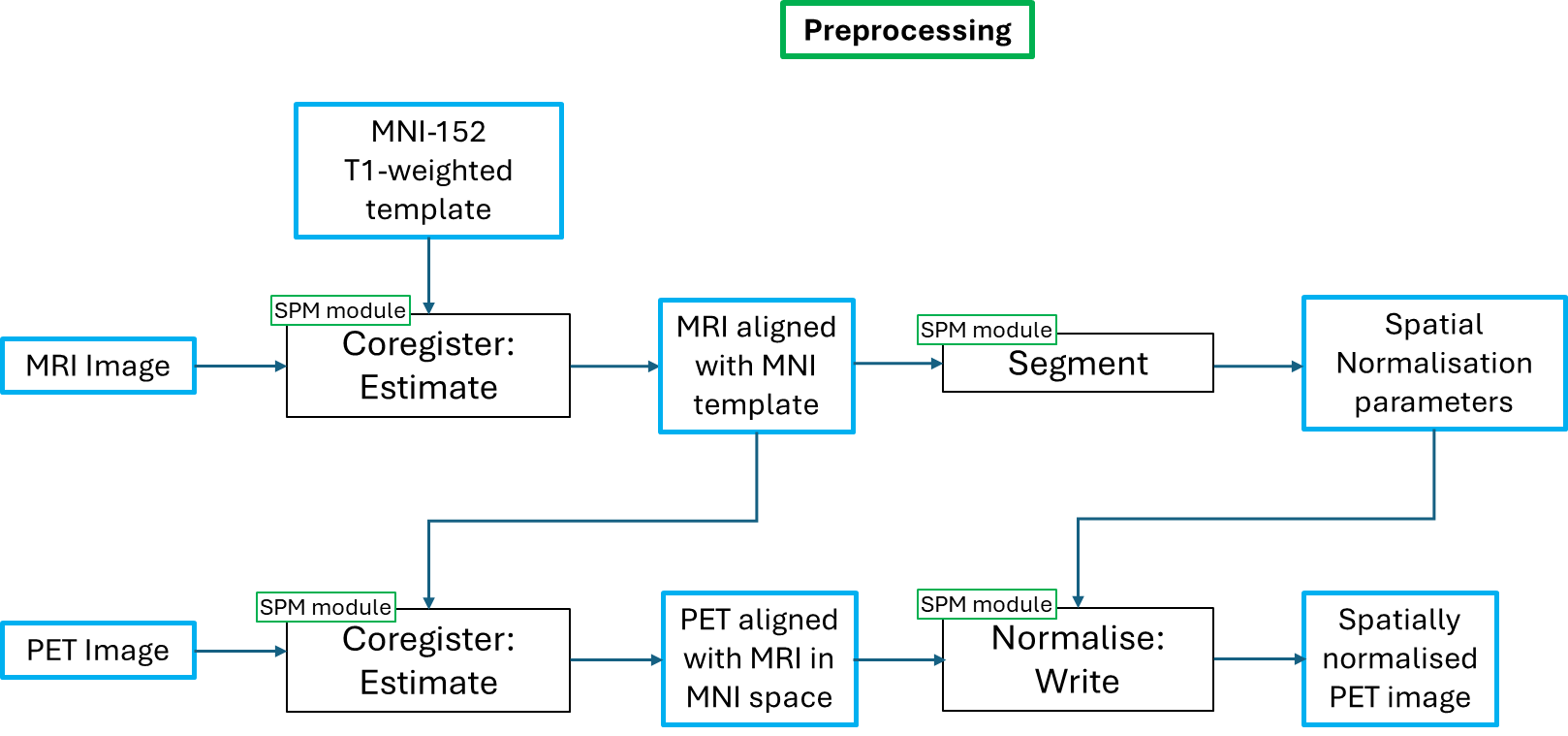


Suppl. Figure 1. Overview of the SPM8 pipeline used to spatially normalise the PET images to the MNI space.


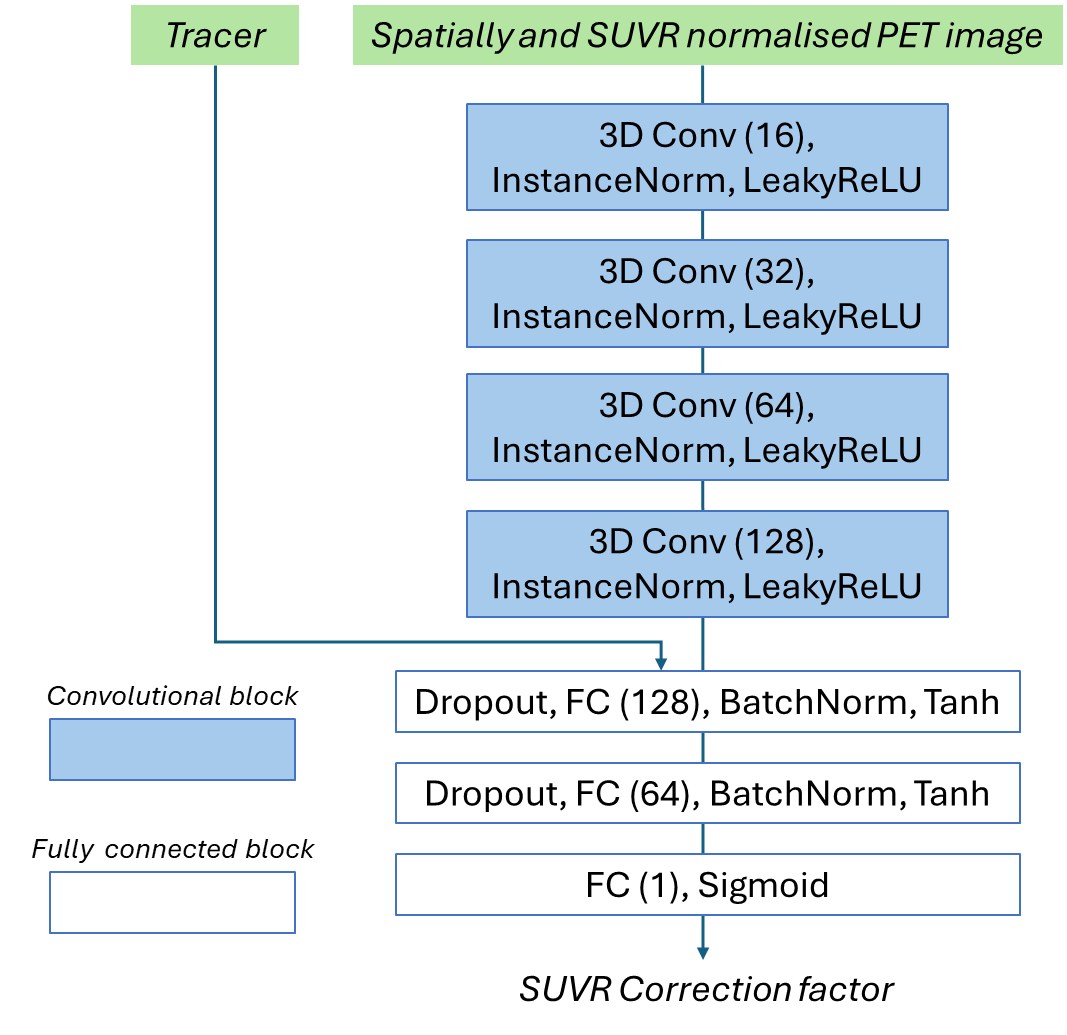


Suppl. Figure 2. Architecture of the DeepSUVR model with 4 convolutional blocks, followed by 3 fully connected layers. The tracer information feeds into the fully connected layer as a one-hot vector


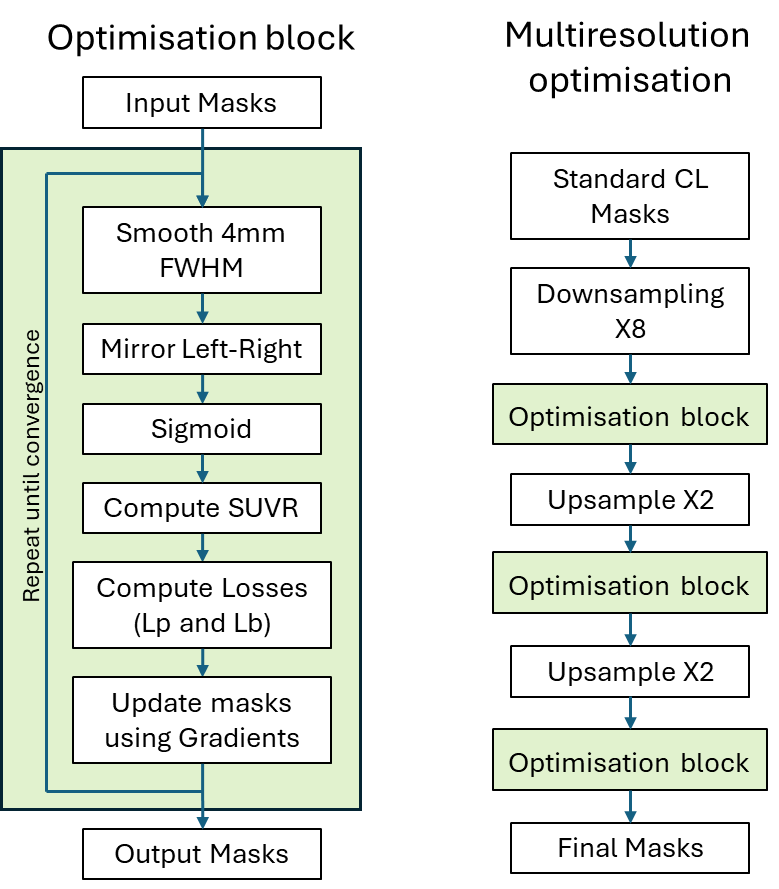


Suppl. Figure 3. Overview of the optimisation procedure employed to derive new mask to match DeepSUVR quantification.


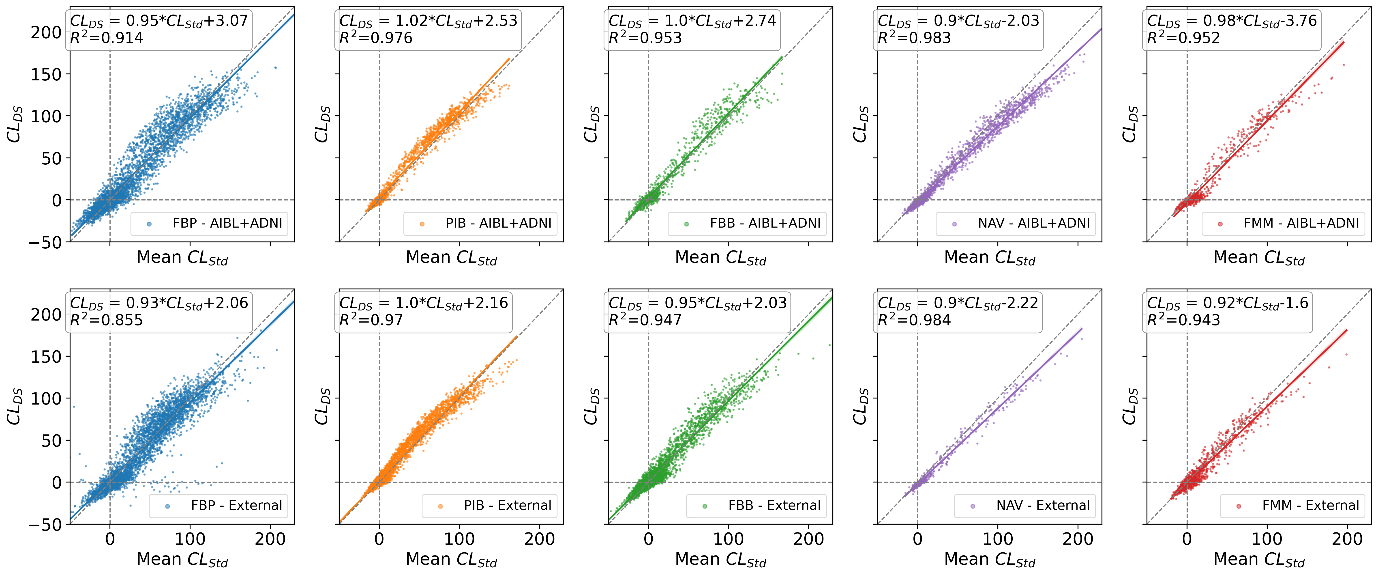


Sup Figure 4. Scatterplot of the correlation between the DeepSUVR Centiloids and the Standard Centiloids. The correlation between the tracers is assessed using the coefficient of determination R^2^. The first row presented the correlation in each tracer in the AIBL+ADNI dataset (FBP=3909, PIB=1747, FBB=902, NAV=2168, FMM=539), which was used to train the model. The bottom row presented the correlation in each tracer in the 10 external datasets (PISA, ADNeT, ADNI DOD, MCSA, OASIS, DLBS, AMYPAD, A4-Learn, HABS-HD, WRAP) - (FBP=3956, PIB=5201, FBB=4935, NAV=448, FMM=1264)


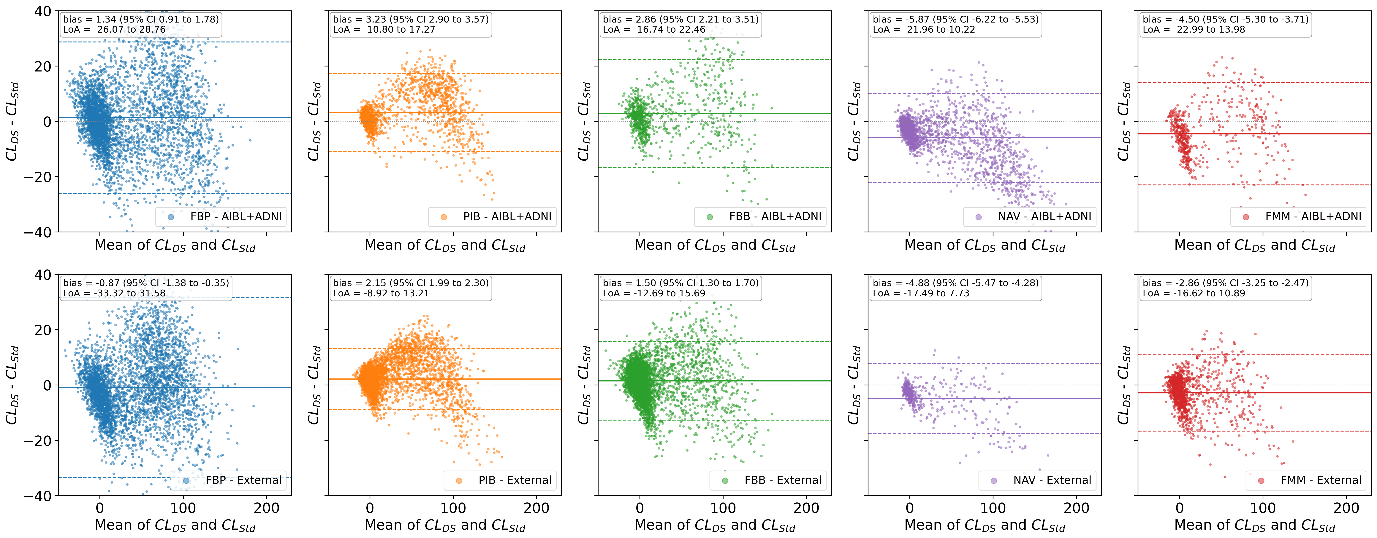
*Sup Figure 5. Bland–Altman plots comparing the Standard and DeepSUVR Centiloids. Each panel shows the mean of the two methods on the x-axis and their difference on the y-axis. The solid line indicates the mean bias, and dashed lines represent the 95% limits of agreement (LoA = bias ± 1.96·SD). The first row presents the comparison in each tracer in the AIBL+ADNI dataset (FBP=3909, PIB=1747, FBB=902, NAV=2168, FMM=539). The bottom row presents the comparison in each tracer in the 10 external datasets (PISA, ADNeT, ADNI DOD, MCSA, OASIS, DLBS, AMYPAD, A4-Learn, HABS-HD, WRAP) - (FBP=3956, PIB=5201, FBB=4935, NAV=448, FMM=1264)*


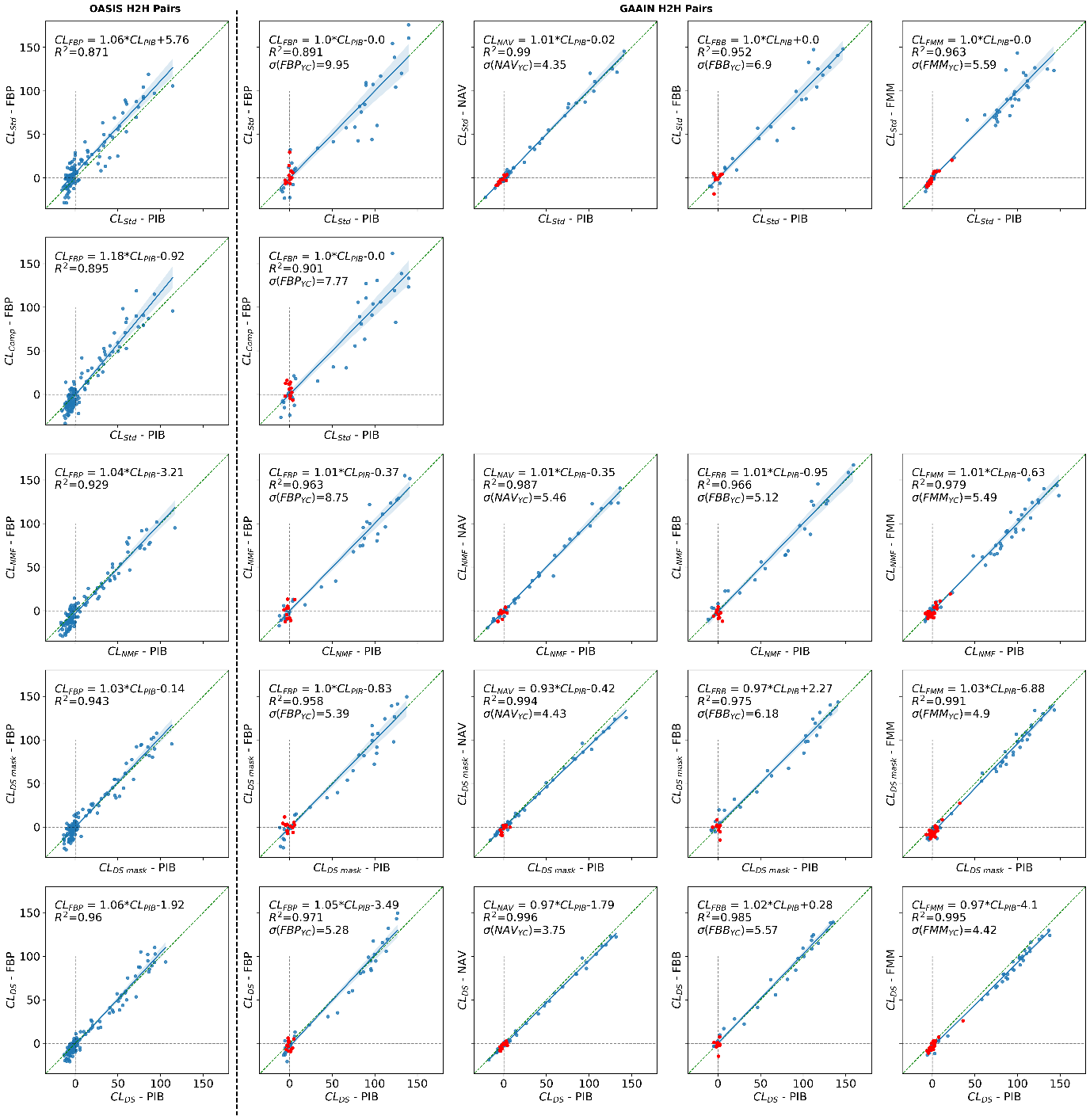


Sup Figure 6. Scatterplot of the PiB vs FBP in the OASIS Head-to-Head dataset (left) and between each 18F-Tracer compared to PIB in the GAAIN Head-to-Head Calibration dataset (Right). The correlation between each pair of tracers is assessed using the coefficient of determination R^2^. Each row shows the results using a different quantification method, with the Standard masks, Composite reference region for FBP, NMF, DeepSUVR-derived masks and DeepSUVR. The standard deviation in the Young Controls is denoted using σ(Tracer_YC_). Note that for FMM, one YC with a CL>20 on both PIB and FMM across all methods was determined to be an outlier and excluded from the standard deviation.


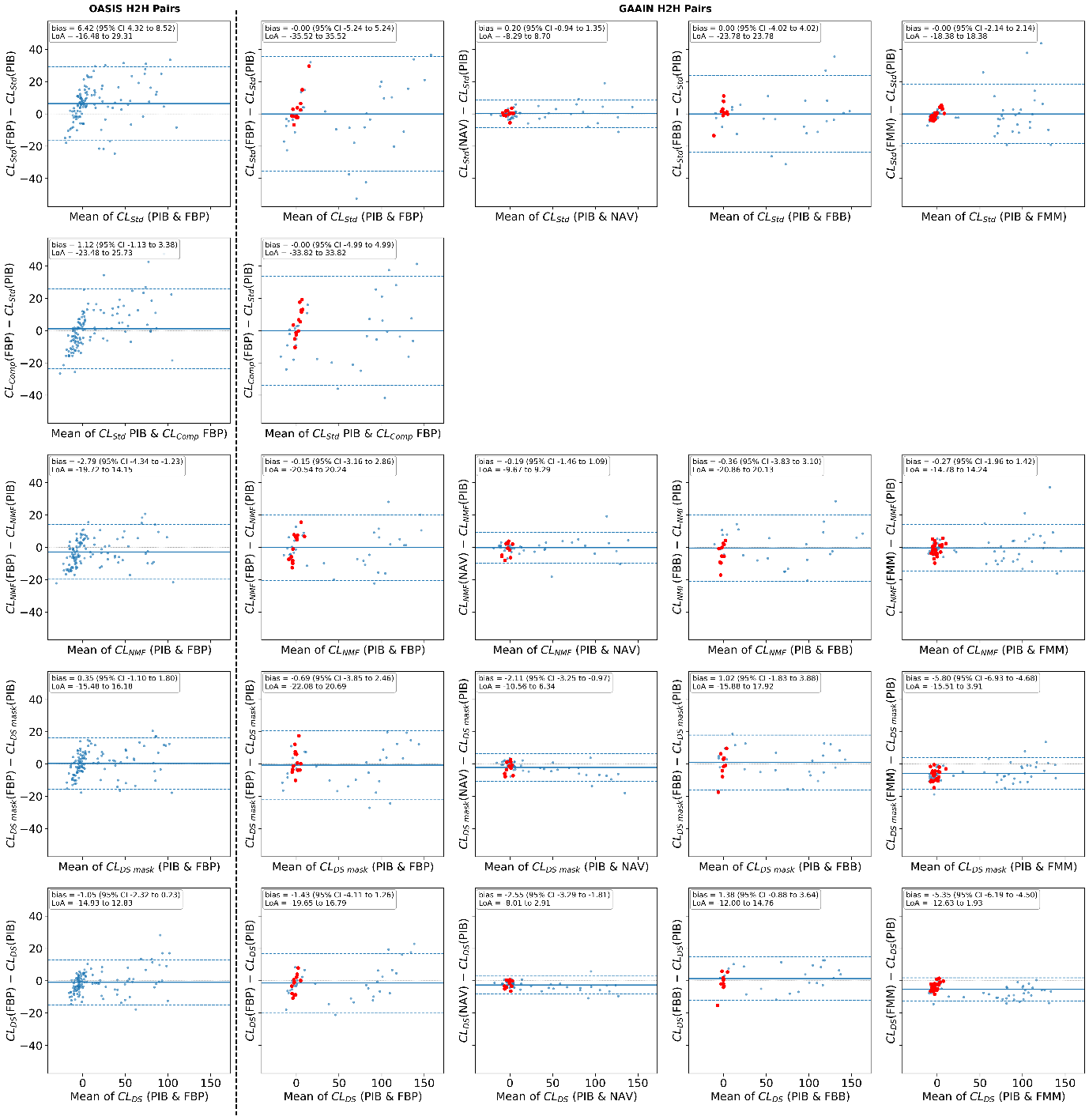


Sup Figure 7. Bland–Altman plots comparing PiB vs FBP in the OASIS Head-to-Head dataset (left) and each 18F tracer versus PiB in the GAAIN Head-to-Head Calibration dataset (right), using Standard masks, Composite reference region for FBP, NMF, DeepSUVR-derived masks and DeepSUVR. Each panel shows the mean of the two methods on the x-axis and their difference on the y-axis. The solid line indicates the mean bias, and dashed lines represent the 95% limits of agreement (LoA = bias ± 1.96·SD). Red points denote Young Control (YC) pairs.


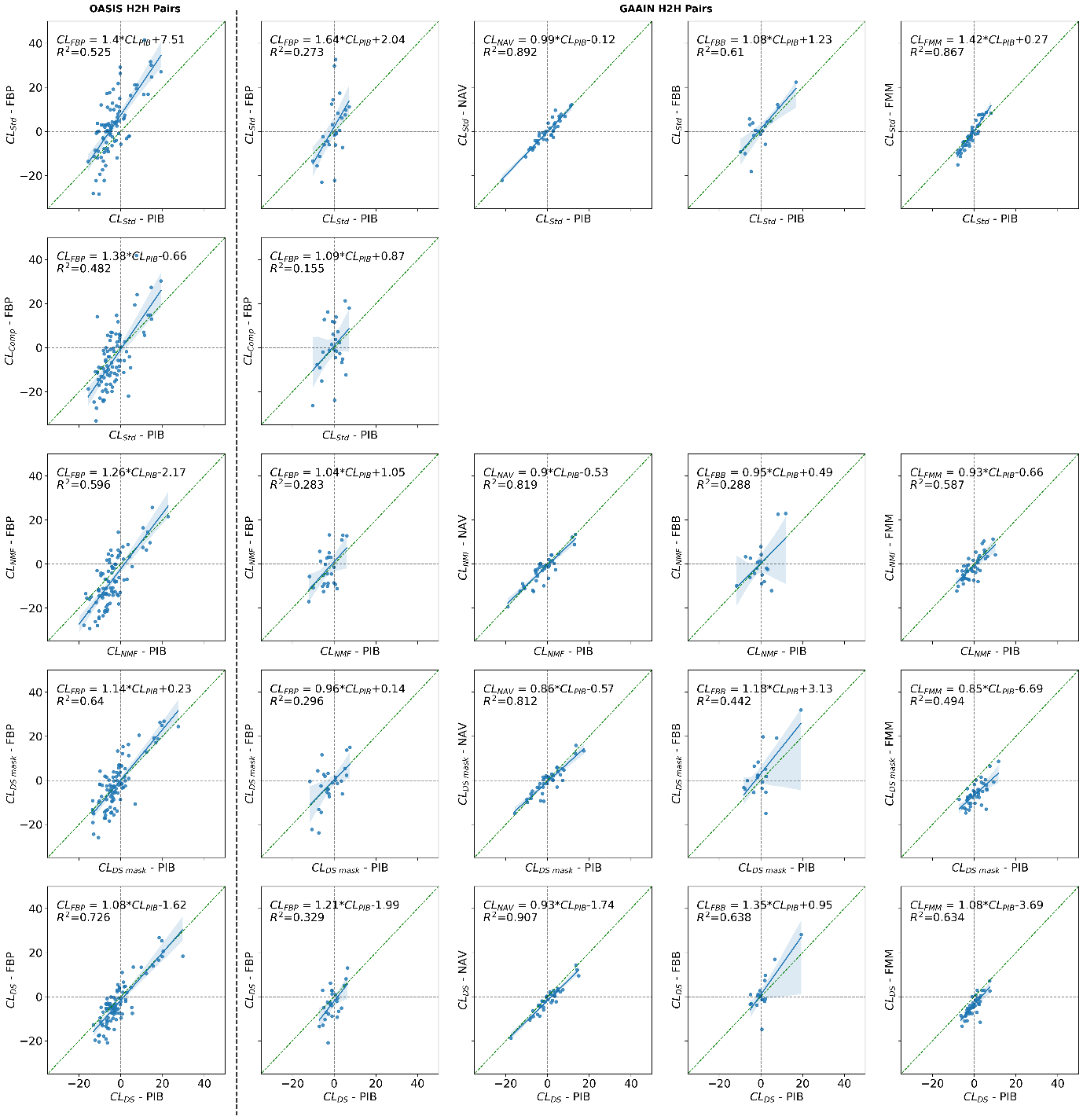


Sup Figure 8. Scatterplot in the PIB CL_Std_<20, of the PIB vs FBP in the OASIS Head-to-Head dataset (left) and between each 18F-Tracer compared to PIB in the GAAIN Head-to-Head Calibration dataset (Right). The correlation between each pair of tracers is assessed using the coefficient of determination R^2^. Each row shows the results using a different quantification method, with the Standard masks, Composite reference region for FBP, NMF, DeepSUVR-derived masks and DeepSUVR.


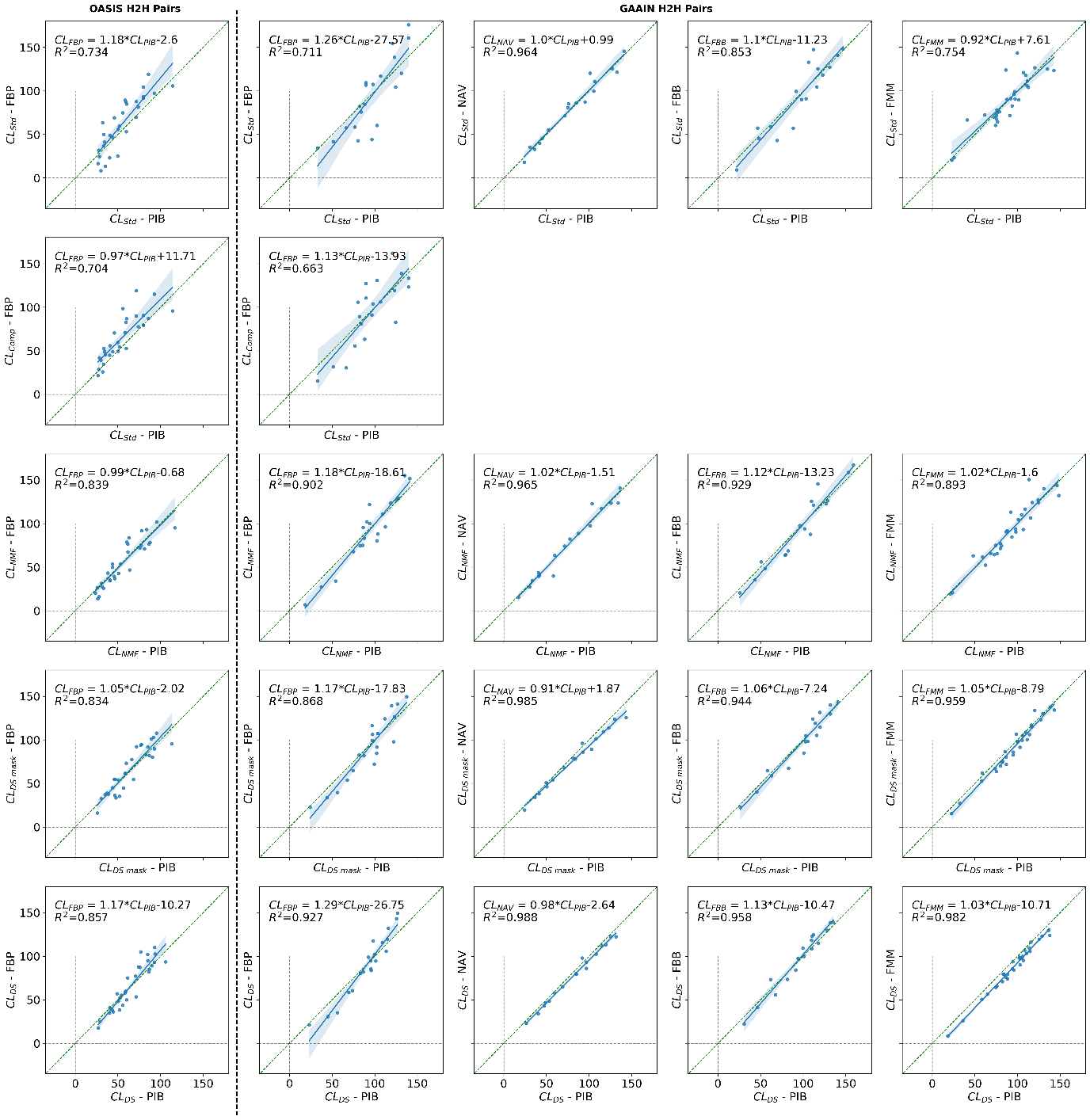


Sup Figure 9. Scatterplot in the PIB CL_Std_≥20, of the PIB vs FBP in the OASIS Head-to-Head dataset (left) and between each 18F-Tracer compared to PIB in the GAAIN Head-to-Head Calibration dataset (Right). The correlation between each pair of tracers is assessed using the coefficient of determination R^2^. Each row shows the results using a different quantification method, with the Standard masks, Composite reference region for FBP, NMF, DeepSUVR-derived masks and DeepSUVR.


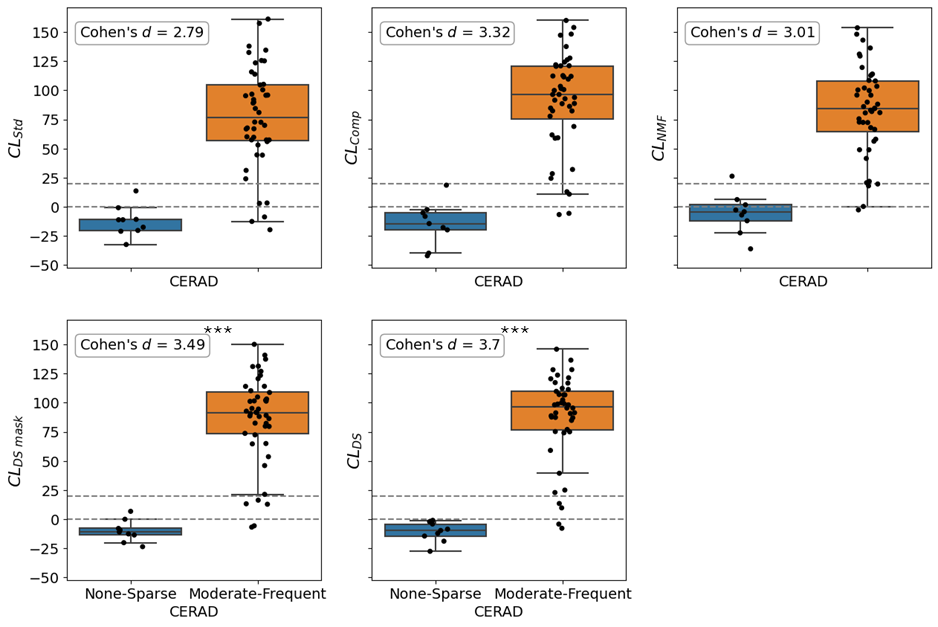


Sup Figure 10. Comparison of FBP CLs in the ADNI Neuropathology datasets between CERAD score for diffuse plaques grouped as none/sparse (N=9) and moderate/frequent (N=44). Effect size (Cohen’s d) and boxplot between none/sparse and moderate/frequent are presented for each quantification method. Horizontal dashed lines indicate the CL=0 and CL=20 thresholds and are presented just for reference. The statistical significance over CL_Std_ based on bootstrapping is indicated using: * p<0.05, **p<0.01, *** p<0.001.


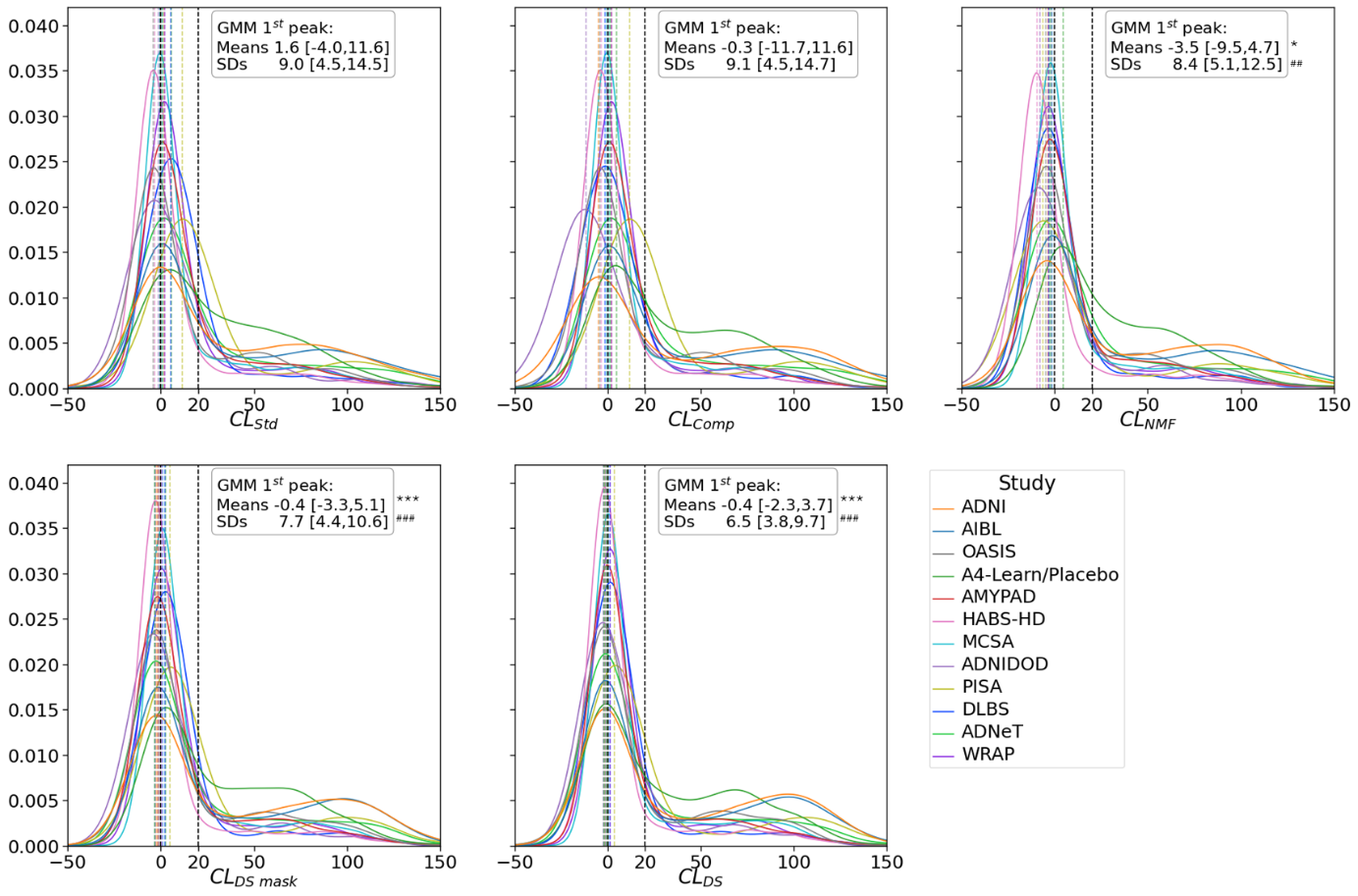


Sup Figure 11. Histogram distribution across the 12 cohorts using the Standard, Composite, NMF, DeepSUVR-derived masks and DeepSUVR CL methods. For each approach, a Gaussian mixture is fitted to the distribution of Cl values of each study, and the mean [min,max] and standard deviation [min,max] of the first peak across all studies is reported. The dashed lines mark the 0CL and 20CL. The methods showing significantly lower variability in the means and standard deviations compared to CL_Std_ across studies based on bootstrapping are indicated using: * p<0.05, ** p<0.01, *** p<0.001 for lower variabilities in the means and ^#^ p<0.05, ^##^ p<0.01, ^###^ p<0.001 for lower variabilities in the standard deviations.


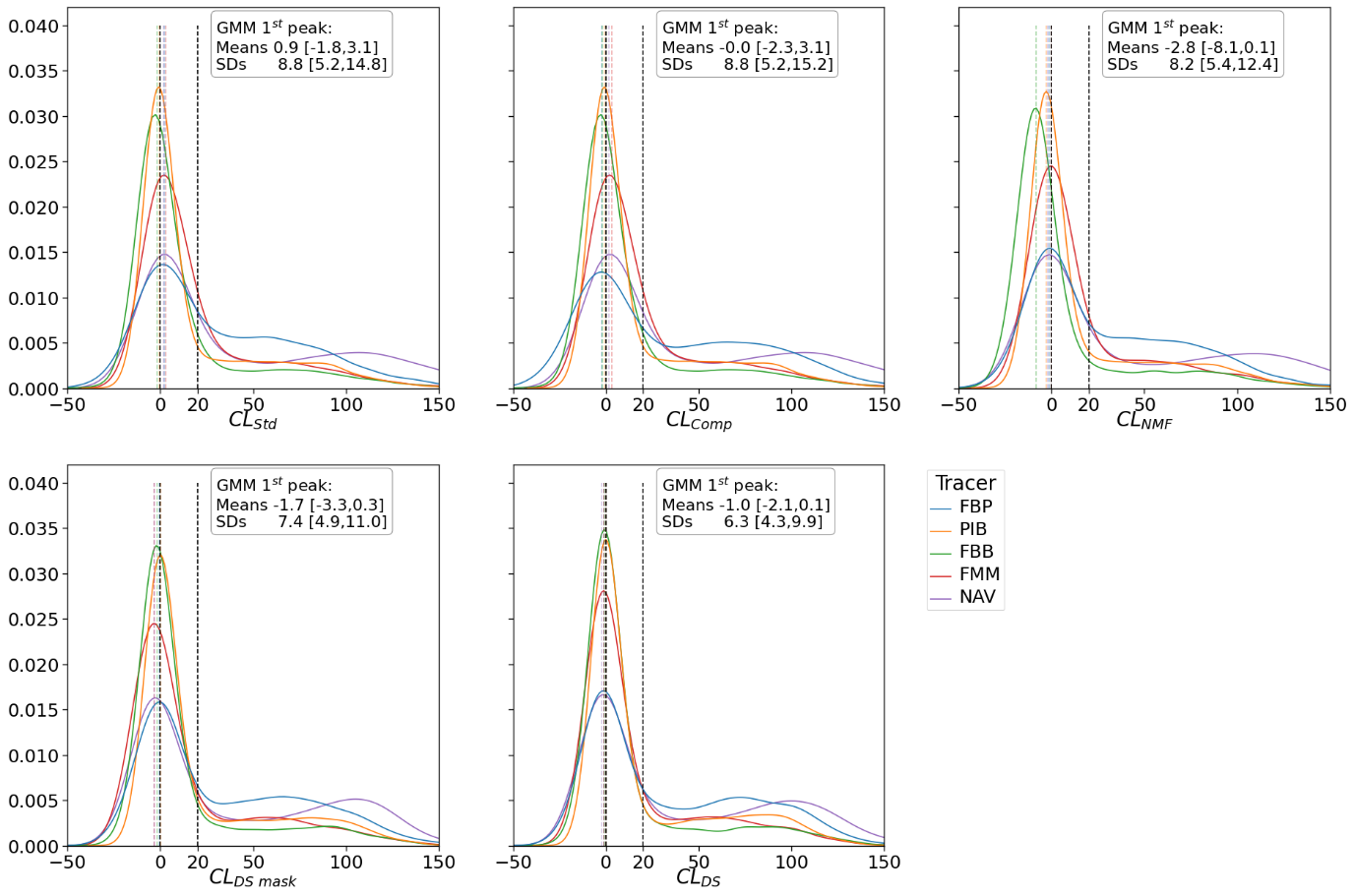


Sup Figure 12. Histogram distribution across the 5 tracers for the 12 cohorts using the Standard, Composite, NMF, DeepSUVR-derived masks and DeepSUVR CL methods. For each approach, a Gaussian mixture is fitted to the distribution of Cl values of each tracer, and the mean [min,max] and standard deviation [min,max] of the first peak across all tracers is reported. The dashed lines mark the 0CL and 20CL. The methods showing significantly lower variability in the means and standard deviations compared to CL_Std_ across tracers based on bootstrapping are indicated using: * p<0.05, ** p<0.01, *** p<0.001 for lower variabilities in the means and ^#^ p<0.05, ^##^ p<0.01, ^###^ p<0.001 for lower variabilities in the standard deviations.


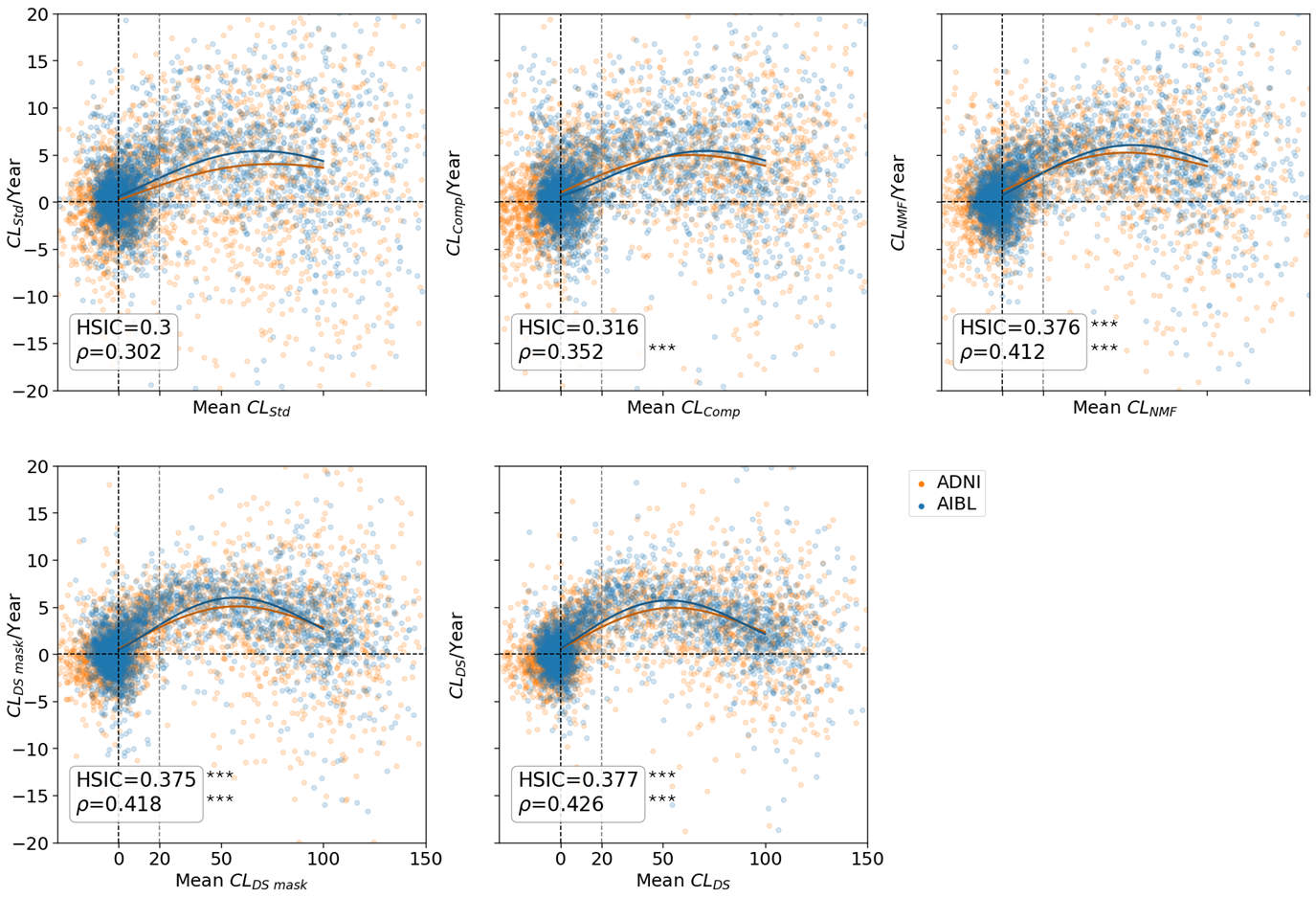


Sup Figure 13. Rate of CL change per year compared to mean CL computed from the 2 training cohorts (AIBL/ADNI) using the Standard, Composite WM, NMF, DeepSUVR-derived mask and DeepSUVR CL methods. Each point represents the mean and rate of change between a pair of consecutive visits from the same participant. Each curve shows a 5th order polynomial fitted to each study. The Spearman rank correlation between Mean CL and CL/Year is denoted using ρ. The vertical dashed lines mark the 0CL and 20CL, while the horizontal dashed line mark the 0CL/Year. The methods showing significantly higher HSIC or Spearman ρ compared to CL_Std_ based on bootstrapping are indicated using: * p<0.05, ** p<0.01, *** p<0.001.


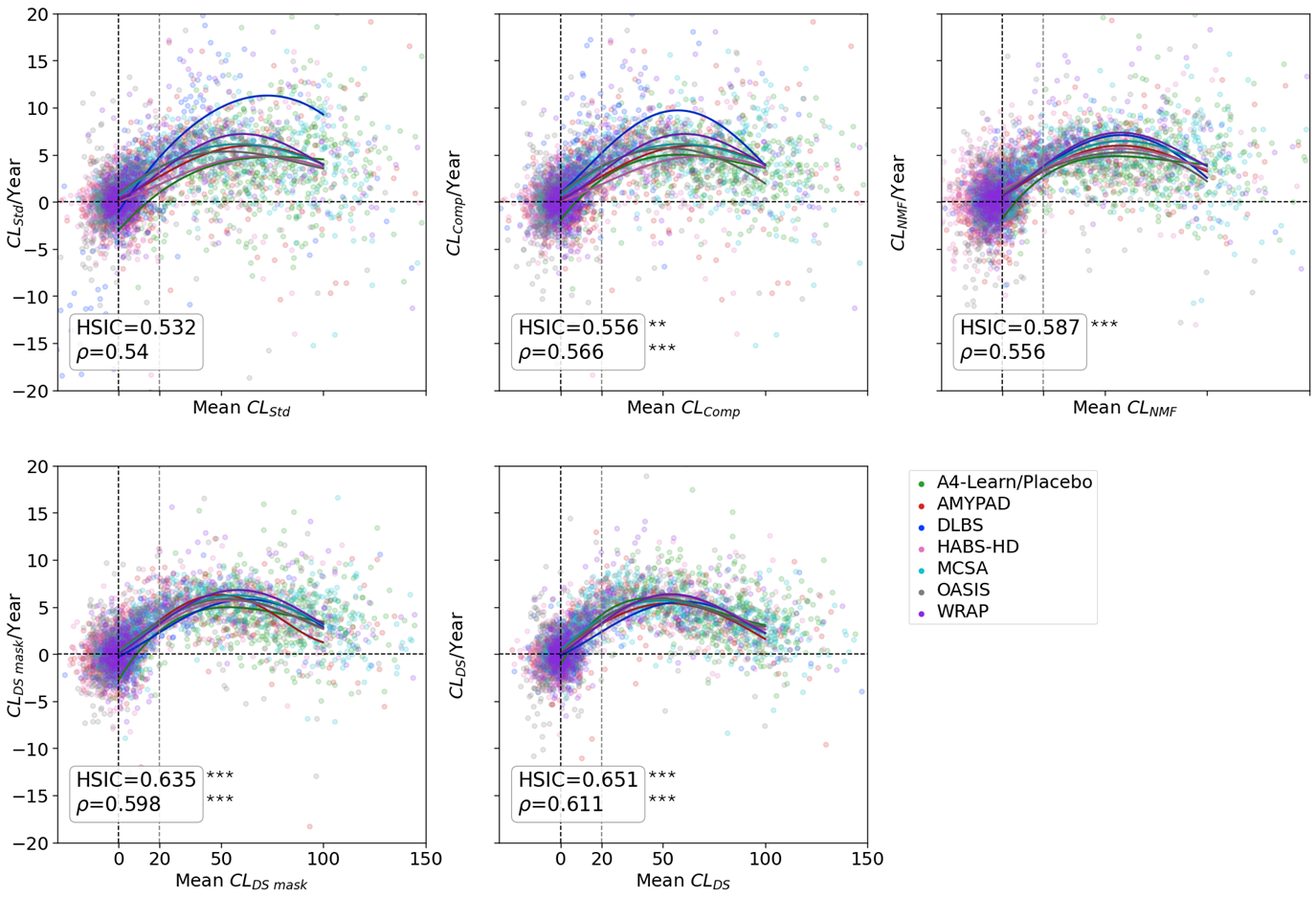


Sup Figure 14. Rate of CL change per year compared to mean CL computed from the 7 testing cohorts with longitudinal data (A4-Placebo, AMYPAD, HABS-HD, OASIS, DLBS, WRAP, MCSA) using the Standard, Composite WM, NMF, DeepSUVR-derived mask and DeepSUVR CL methods. Each point represents the mean and rate of change between a pair of consecutive visits from the same participant. Each curve shows a 5th order polynomial fitted to each study. The Spearman rank correlation between Mean CL and CL/Year is denoted using ρ. The vertical dashed lines mark the 0CL and 20CL, while the horizontal dashed line mark the 0CL/Year. The methods showing significantly higher HSIC or Spearman ρ compared to CL_Std_ based on bootstrapping are indicated using: * p<0.05, ** p<0.01, *** p<0.001.

*
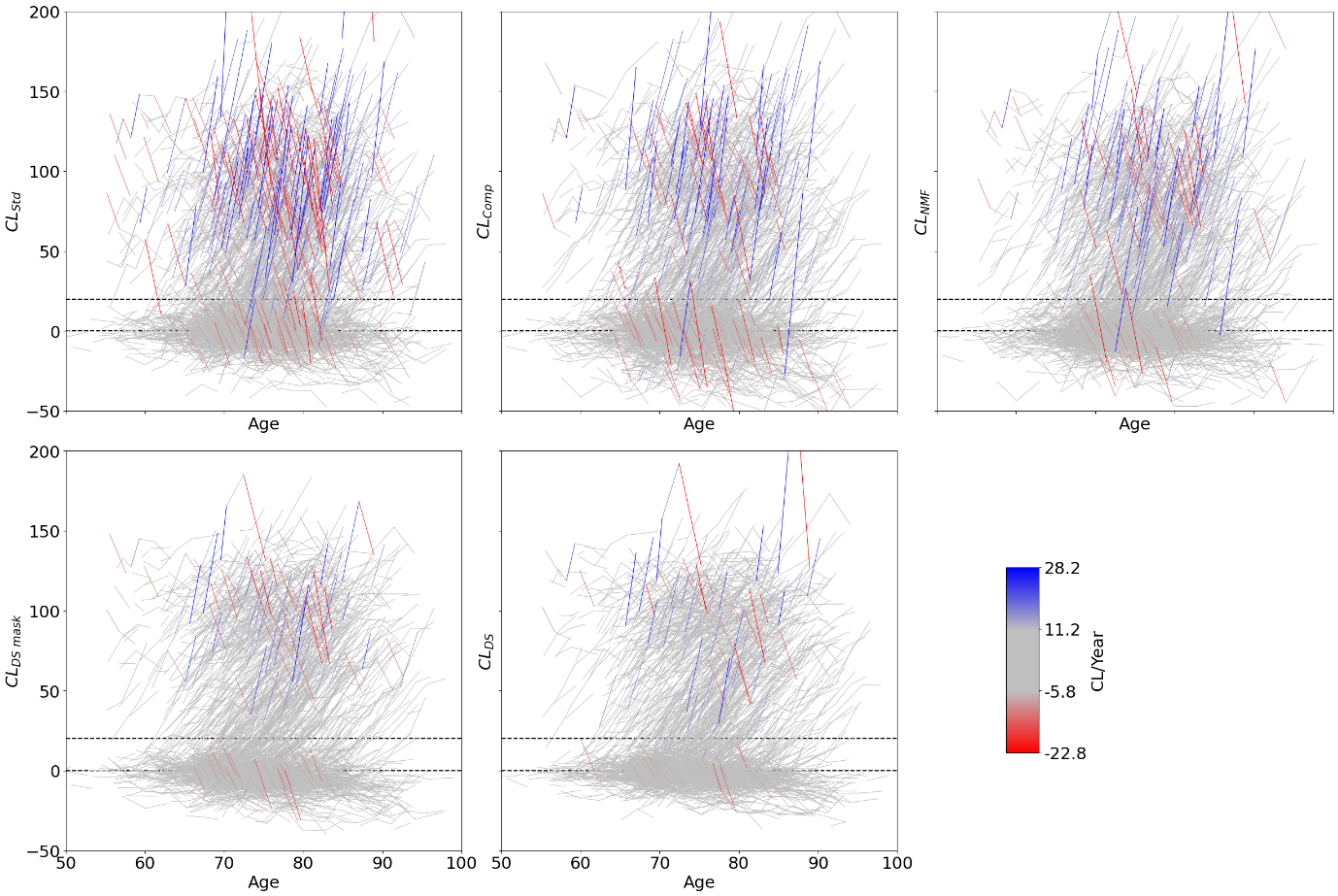
*

Sup Figure 15. Spaghetti plot of Centiloid value changes over time of all subjects with longitudinal scans in the training set. Each line connects CL values from unique subjects. Trajectories in red indicate negative changes that are greater than -5.8CL/Y and trajectories in blue indicate positive changes that are greater than 11.2CL/Y. The horizontal dashed lines mark 0CL and 20CL.


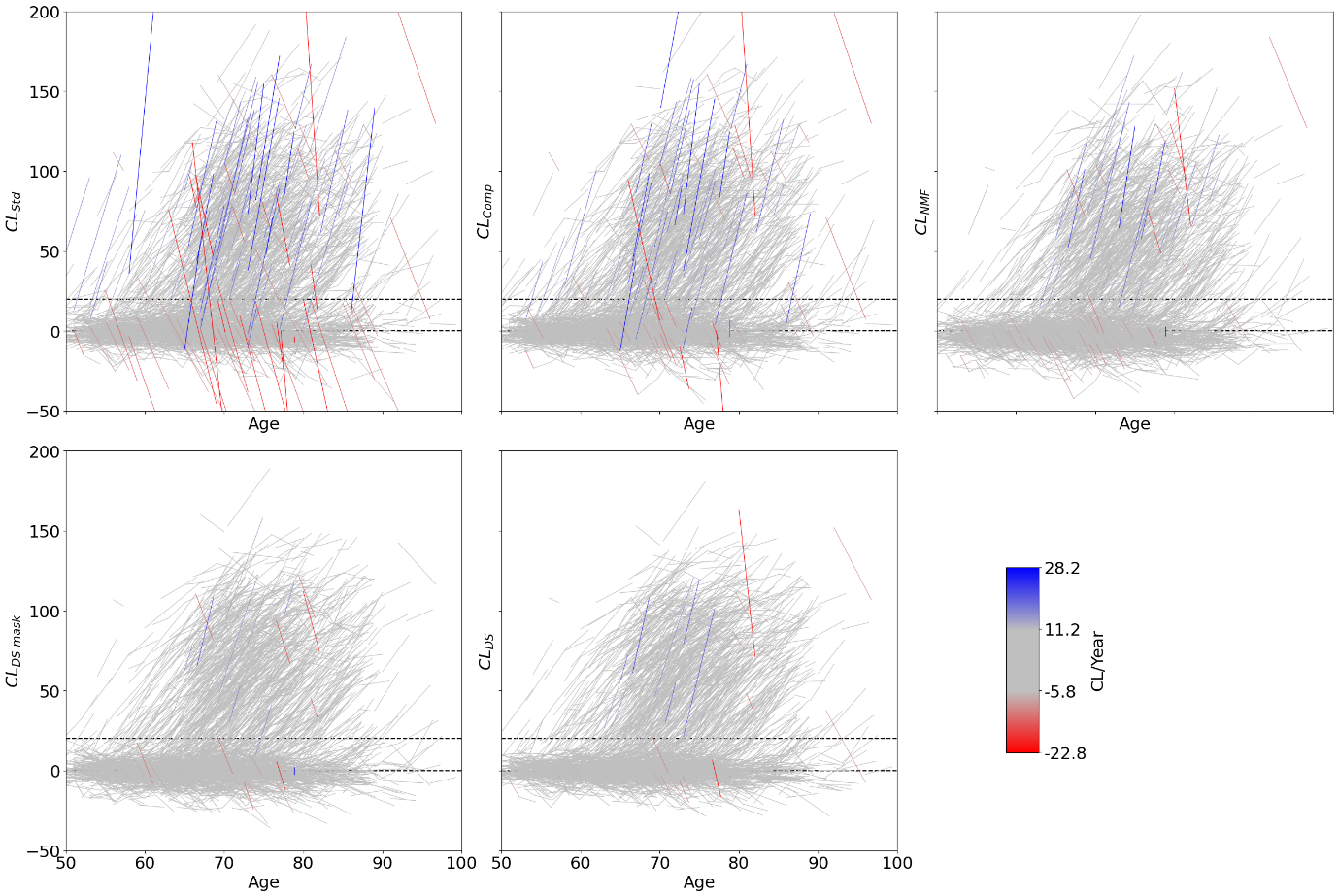


Sup Figure 16. Spaghetti plot of Centiloid value changes over time of all subjects with longitudinal scans in the testing set. Each line connects CL values from unique subjects. Trajectories in red indicate negative changes that are greater than -5.8CL/Y and trajectories in blue indicate positive changes that are greater than 11.2CL/Y. The horizontal dashed lines mark 0CL and 20CL.


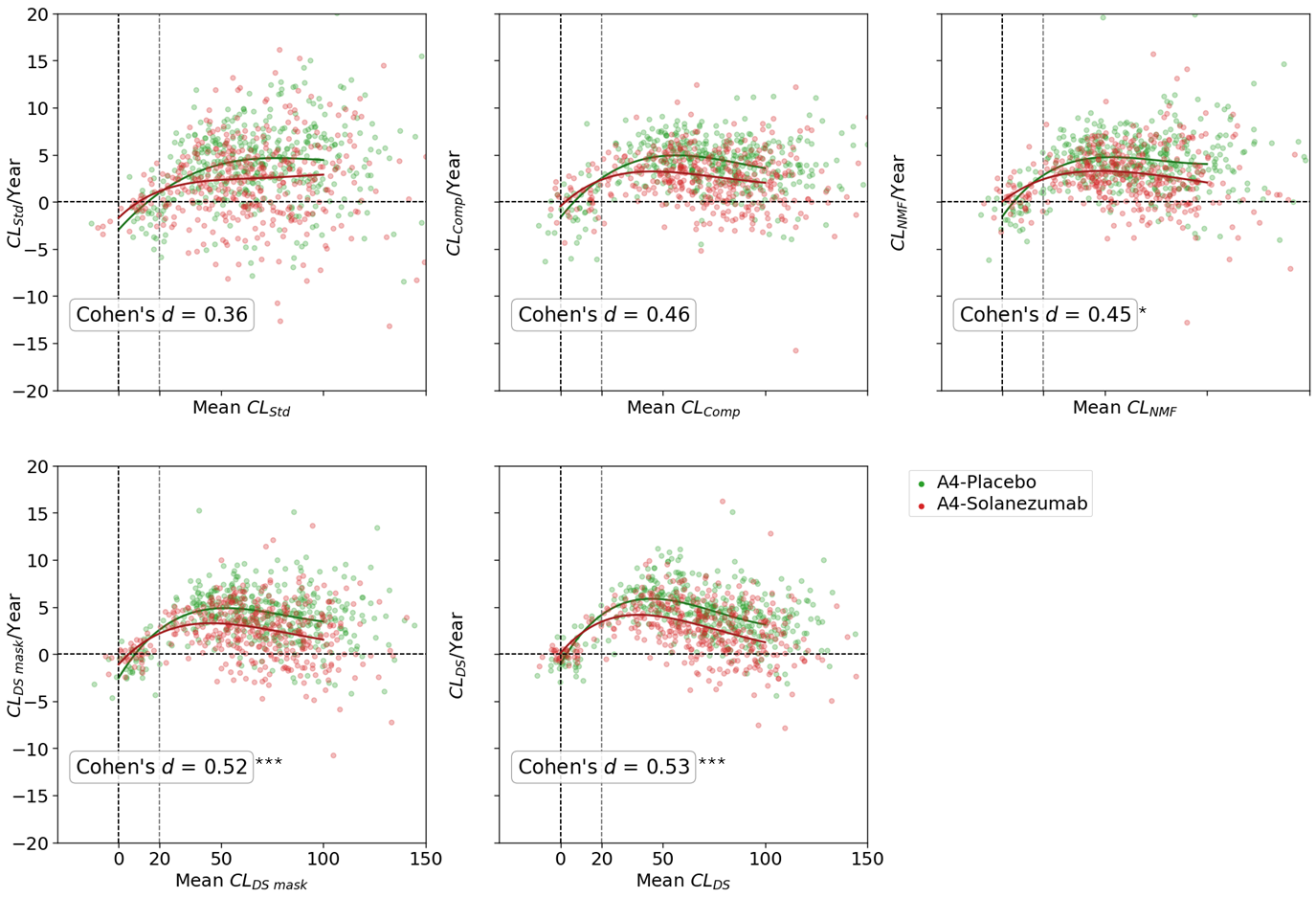


Sup Figure 17. Rate of CL change per year compared to mean CL computed from the placebo and treatment arms of the A4 Study Standard, Composite WM, NMF, DeepSUVR-derived mask and DeepSUVR CL methods. Each point represents the mean and rate of change between the 2 and 56 months visits from the same participant. Each curve shows a 5th order polynomial fitted to each study. The effect size of the CL accumulation per year between the 2 arms of the A4 study is denoted using Cohen’s d. The vertical dashed lines mark the 0CL and 20CL, while the horizontal dashed line mark the 0CL/Year. The methods showing significantly higher Cohen’s d compared to CL_Std_ based on bootstrapping are indicated using: * p<0.05, ** p<0.01, *** p<0.001.


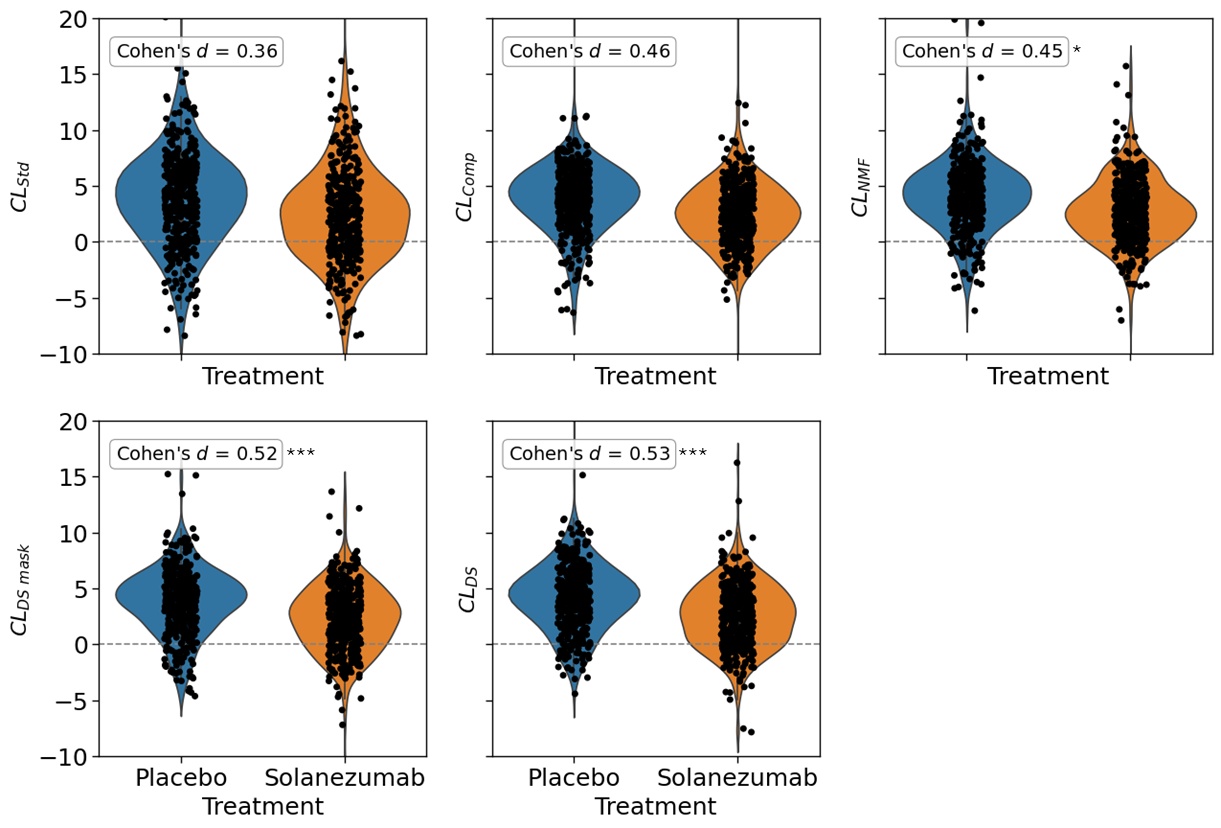


Sup Figure18. Violin plot of the rate of CL change per year in the placebo and treatment arms of the A4 Study Standard, Composite WM, NMF, DeepSUVR-derived mask and DeepSUVR CL methods. The effect size of the CL accumulation per year between the 2 arms of the A4 study is denoted using Cohen’s d. The horizontal dashed lines marks 0CL/Y and 20CL, while the horizontal dashed line mark the 0CL/Year. The methods showing significantly higher Cohen’s d compared to CL_Std_ based on bootstrapping are indicated using: * p<0.05, ** p<0.01, *** p<0.001.


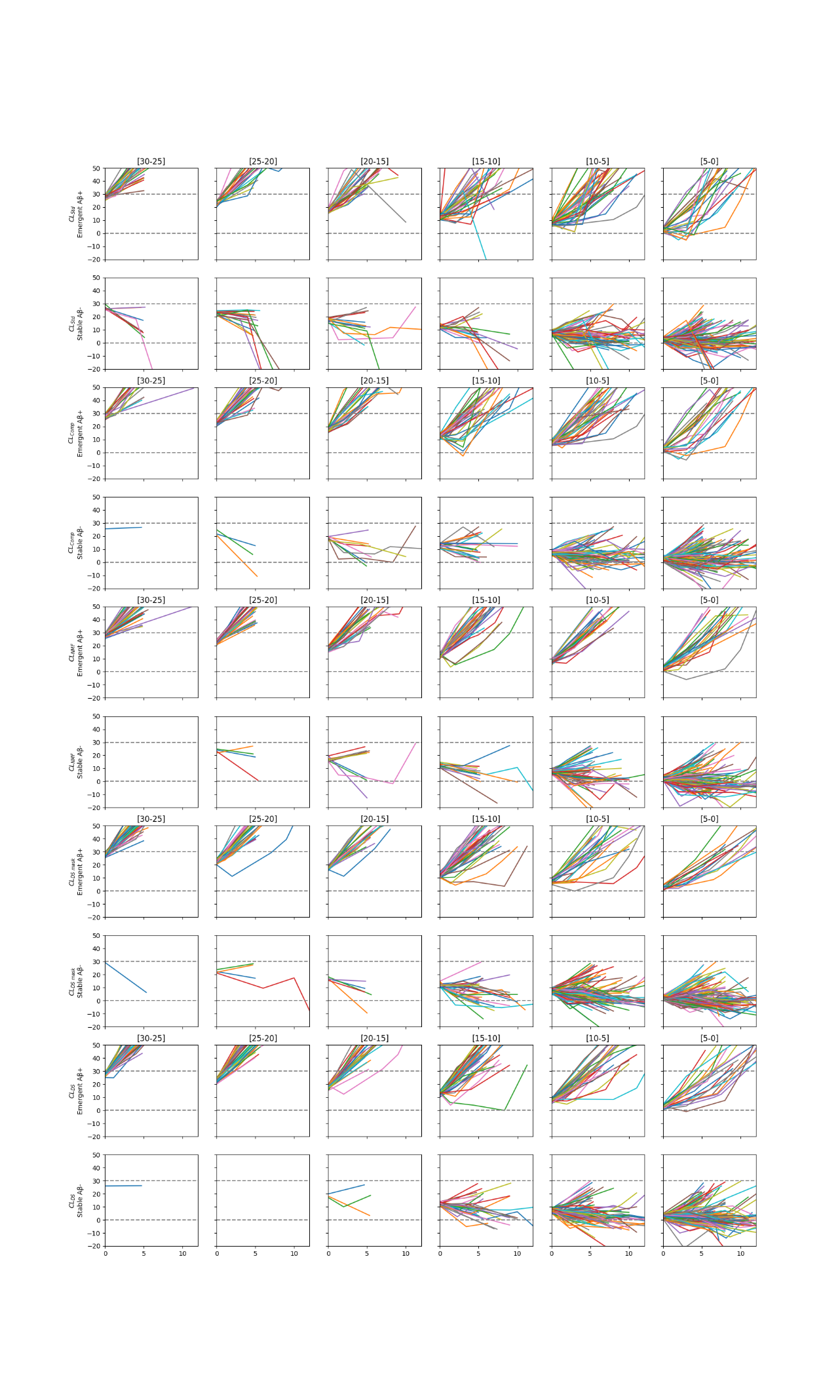


Sup Figure 19. Spaghetti plot of Centiloid changes against the number of years since baseline in the testing set with each column representing a baseline CL bracket. For each quantification method, the top row only shows the emergent Aβ+, defined as participants with a negative baseline CL (<30CL) and with at least one follow-up above 30CL. The second row only shows participants which remain stable Aβ-. The horizontal dashed lines mark 0CL and 30CL.


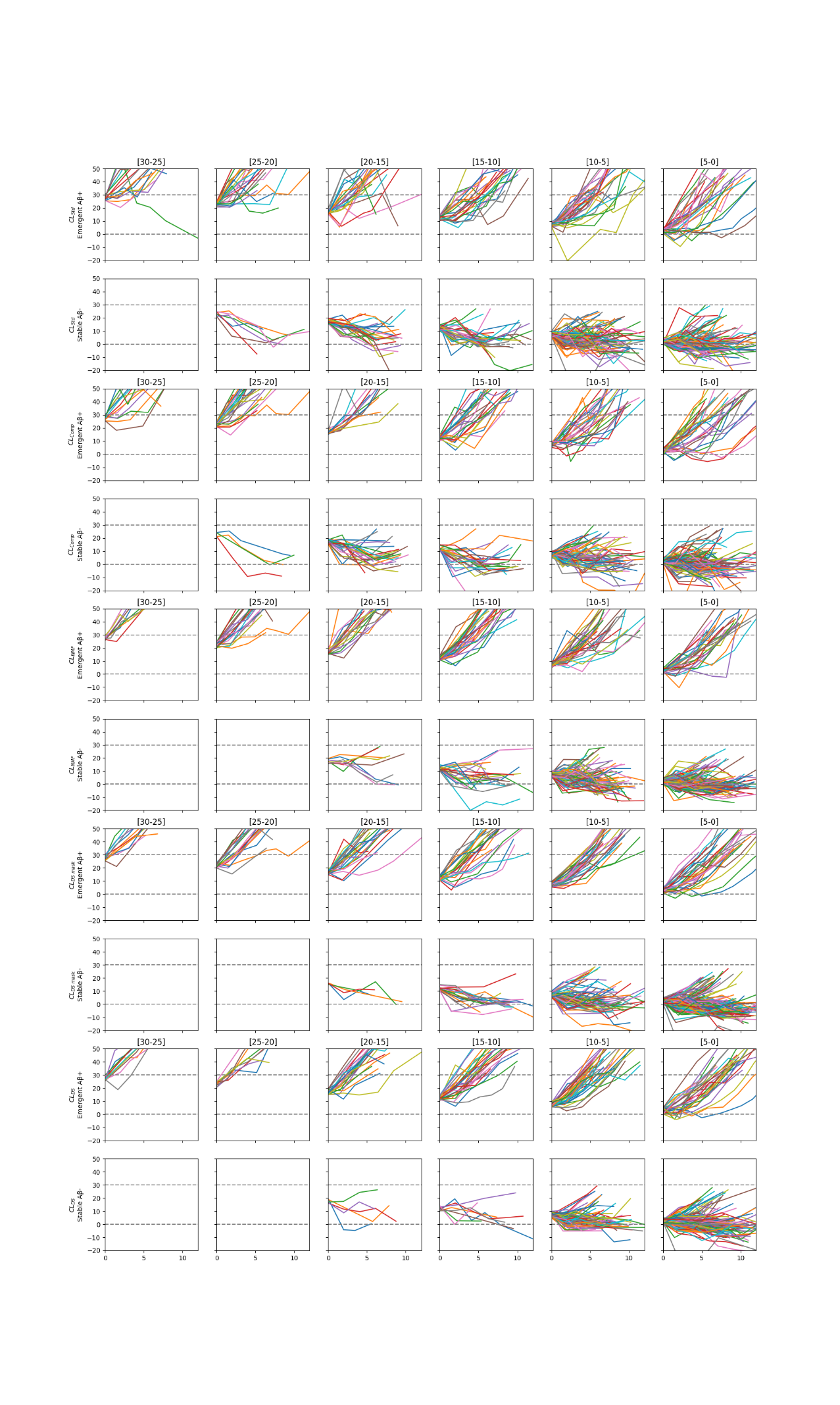


Sup Figure 20. Spaghetti plot of Centiloid changes against the number of years since baseline in the training set with each column representing a baseline CL bracket. For each quantification method, the top row only shows the emergent Aβ+, defined as participants with a negative baseline CL (<30CL) and with at least one follow-up above 30CL. The second row only shows participants which remain stable Aβ-. The horizontal dashed lines mark 0CL and 30CL.


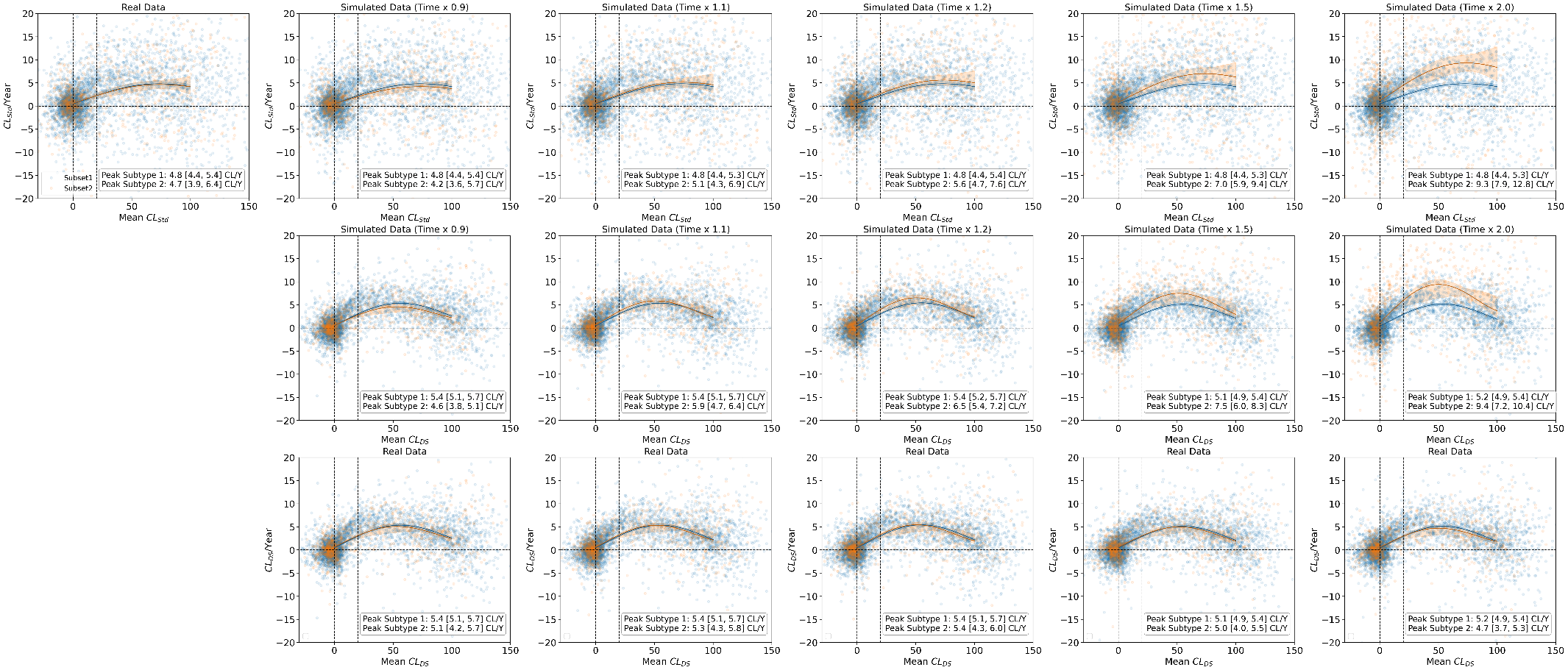


Sup Figure 21. Simulated slowed/accelerated rate of CL increase, where 20% of participants (Subset 2) in the training set are assigned a 0.9x, 1.1x, 1.2x, 1.5x and 2x increase rate of CL change before training a new DeepSUVR model (top row). Evaluation of each DeepSUVR model on the out-of-fold simulated data (middle row) and on the real data with no simulated differences in trajectories (bottom row). Each column shows the simulated training dataset, and the inference of the corresponding DeepSUVR model (trained on that simulated dataset) on the out-of-fold simulated and real dataset. Each curve shows a 5th order polynomial fitted to each subset, with the peak of the fitted polynomial used as an estimate of the peak rate of accumulation with confidence intervals computed using bootstrap.


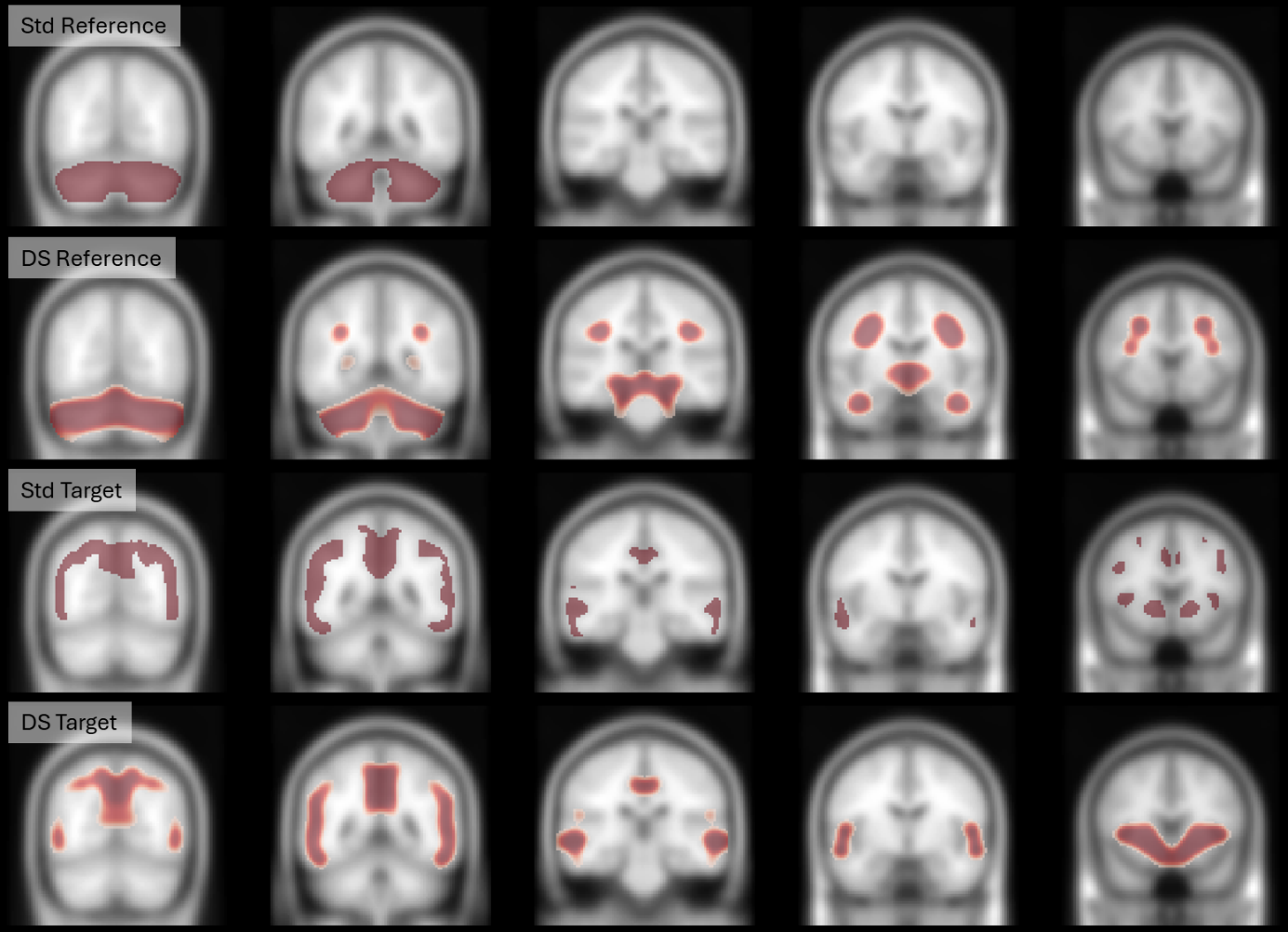


Suppl. Figure 22. Coronal views of the Standard CL reference mask (row1) and the new reference mask derived from DeepSUVR (row2), the Standard CL target mask (row3) and the new target mask derived from DeepSUVR (row4)


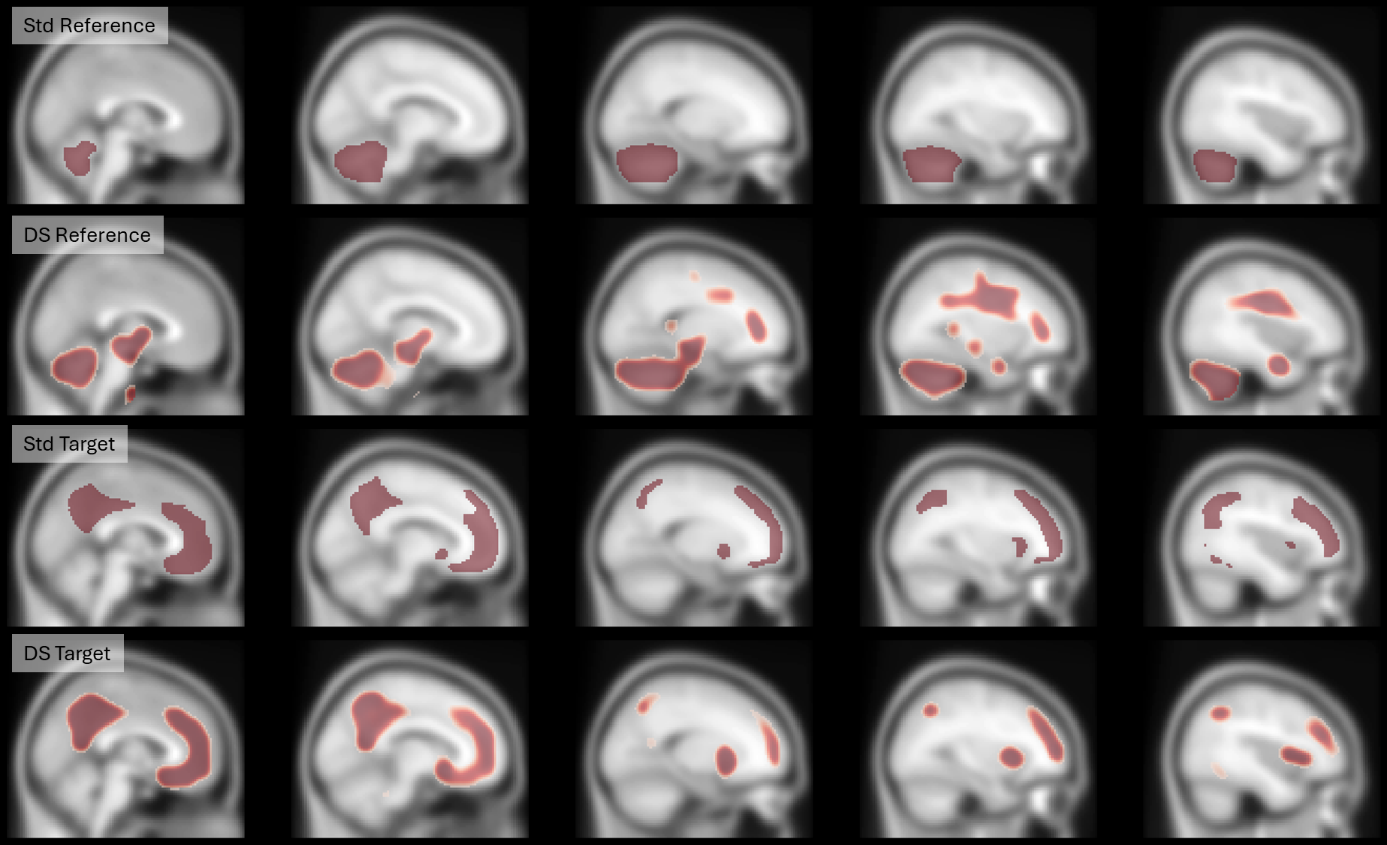


Suppl. Figure 23. Sagittal views of the Standard CL reference mask (row1) and the new reference mask derived from DeepSUVR (row2), the Standard CL target mask (row3) and the new target mask derived from DeepSUVR (row4)


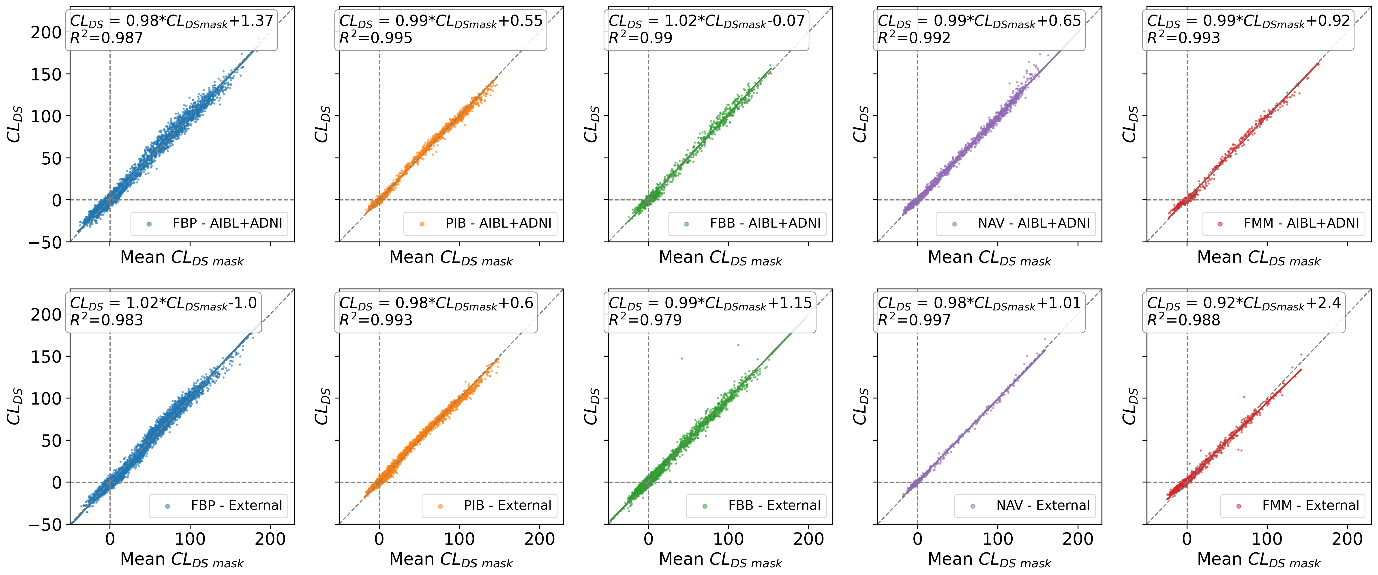


Sup Figure 24. Scatterplot of the correlation between the DeepSUVR Centiloids and the Centiloids obtained using the DeepSUVR-derived masks. The correlation between the tracers is assessed using the coefficient of determination R^2^. The first row presented the correlation in each tracer in the AIBL+ADNI dataset, which was used to construct the new masks. The bottom row presented the correlation in each tracer in the 10 external datasets (PISA, ADNeT, ADNI DOD, MCSA, OASIS, DLBS, AMYPAD, A4-Learn, HABS-HD, WRAP)


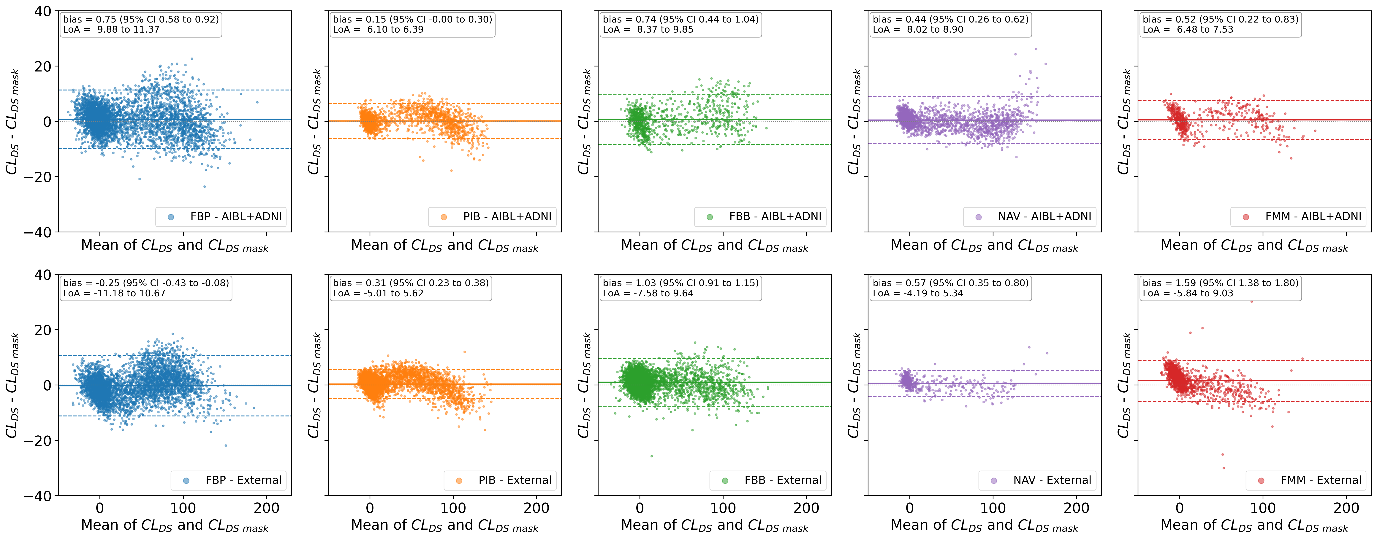


Sup Figure 25. Bland–Altman plots comparing DeepSUVR Centiloids and the Centiloids obtained using the DeepSUVR-derived masks. Each panel shows the mean of the two methods on the x-axis and their difference on the y-axis. The solid line indicates the mean bias, and dashed lines represent the 95% limits of agreement (LoA = bias ± 1.96·SD). The first row presents the comparison in each tracer in the AIBL+ADNI dataset, which was used to construct the new masks. The bottom row presents the comparison in each tracer in the 10 external datasets (PISA, ADNeT, ADNI DOD, MCSA, OASIS, DLBS, AMYPAD, A4-Learn, HABS-HD, WRAP)

|  | AIBL | ADNI | A4-Learn | ADNI-DOD | AMYPAD | DLBS | HABS-HD |
| --- | --- | --- | --- | --- | --- | --- | --- |
| *Baseline Age* | 72.18 (7.98) | 73.02 (7.63) | 71.66 (4.76) | 69.91 (4.72) | 68.23 (9.08) | 64.89 (13.44) | 65.47 (8.68) |
| *Baseline CDR* | 0.23 (0.35) | 0.35 (0.35) | 0.0 (0.02) | 0.1 (0.32) | NA | NA | 0.18 (0.38) |
| *Baseline MMSE* | 27.18 (3.08) | 27.57 (2.82) | 28.81 (1.23) | 28.3 (1.61) | NA | 28.33 (1.23) | 22.46 (224.95) |
| *Length of follow-up* | 5.3 (3.43) | 4.63 (2.91) | 4.68 (0.92) | 1.09 (0.22) | 3.31 (1.25) | 5.81 (2.66) | 2.16 (0.28) |
| *Number of longitudinal studies* | 1202 | 1132 | 925 | 9 | 784 | 176 | 608 |
| *Number of scans per tracer (FBP/PIB/FBB/FMM/NAV)* | 652/1545/243/539/2169 | 3257/202/659/-/- | 2706/-/-/-/- | 243/-/-/-/- | -/-/999/1265/- | 542/-/-/-/- | -/-/3685/-/- |
| Number of scanner models | 5 | 27 | NA | 15 | 21 | 3 | 2 |
| Number of visits | 3.44 (1.64) | 3.07 (1.31) | 2.0 (0.0) | 2.0 (0.0) | 2.15 (0.36) | 2.4 (0.49) | 2.0 (0.0) |
|  |  |  |  |  |  |  |  |
|  | MCSA | OASIS | WRAP | ADNeT | PISA | All | |
| *Baseline Age* | 70.84 (9.95) | 69.4 (9.51) | 64.81 (7.74) | 67.13 (7.19) | 61.8 (6.97) | 69.62 (8.79) | |
| *Baseline CDR* | 0.07 (0.18) | 0.12 (0.27) | NA | 0.1 (0.22) | NA | 0.16 (0.31) | |
| *Baseline MMSE* | 28.12 (1.73) | 28.49 (2.19) | NA | 27.81 (2.63) | NA | 26.96 (97.97) | |
| *Length of follow-up* | 3.38 (1.72) | 5.51 (3.28) | 6.45 (4.18) | NA | NA | 4.37 (2.73) | |
| *Number of longitudinal studies* | 829 | 400 | 317 | 0 | 0 | 6382 | |
| *Number of scans per tracer (FBP/PIB/FBB/FMM/NAV)* | -/2989/-/-/- | 466/1098/-/-/- | -/1114/-/-/19 | -/-/-/-/429 | -/-/251/-/- | 7866/6948/5828/1814/2617 | |
| Number of scanner models | 2 | 4 | 3 | 3 | 1 | - | |
| Number of visits | 2.44 (0.7) | 2.94 (1.14) | 2.91 (1.09) | NA | NA | 2.65 (1.16) | |

Sup Table 1. Basic demographics, with mean (standard deviation) and distribution of the number of scans per tracers used in each cohort.

|  | AIBL+ADNI Training | AIBL+ADNI Testing | External Testing |
| --- | --- | --- | --- |
| *Baseline Age* | 72.55 (6.99) | 72.6 (8.77) | 68.43 (8.88) |
| *Baseline CDR* | 0.23 (0.32) | 0.4 (0.38) | 0.09 (0.26) |
| *Baseline MMSE* | 27.92 (2.47) | 26.52 (3.42) | 26.77 (117.93) |
| *Length of follow-up* | 5.03 (3.26) | 4.31 (2.32) | 4.03 (2.34) |
| *Number of longitudinal studies* | 2129 | 204 | 4052 |
| *Number of scans per tracer (FBP/PIB/FBB/FMM/NAV)* | 3204/1607/451/491/1396 | 705/140/451/48/772 | 3957/5201/4935/1265/448 |
| Number of visits | 3.36 (1.53) | 2.23 (0.45) | 2.3 (0.69) |

Sup Table 2. Basic demographics of the AIBL/ADNI training and testing set and external testing set, with mean (standard deviation) and distribution of the number of scans per tracers used in each cohort.

|  | Subset 1 | Subset 2 | Subset 3 | Subset 4 | Subset 5 |
| --- | --- | --- | --- | --- | --- |
| *Baseline Age* | 72.25 (7.47) | 72.6 (7.01) | 71.86 (6.55) | 72.99 (7.05) | 73.04 (6.78) |
| *Baseline CDR* | 0.24 (0.34) | 0.21 (0.29) | 0.24 (0.28) | 0.24 (0.31) | 0.24 (0.38) |
| *Baseline MMSE* | 27.97 (2.27) | 27.99 (2.46) | 28.09 (2.16) | 27.79 (2.45) | 27.76 (2.93) |
| *Length of follow-up* | 5.07 (3.48) | 4.95 (3.11) | 5.09 (3.33) | 4.96 (3.2) | 5.08 (3.18) |
| *Number of longitudinal studies* | 436 | 414 | 417 | 422 | 440 |
| *Number of scans per tracer (FBP/PIB/FBB/FMM/NAV)* | 641/332/117/91/286 | 649/304/84/90/269 | 624/310/75/99/292 | 645/305/79/100/260 | 645/356/96/111/289 |
| Number of visits | 3.36 (1.64) | 3.37 (1.49) | 3.36 (1.54) | 3.29 (1.47) | 3.4 (1.5) |

Sup Table 3. Basic demographics of each subset of the AIBL/ADNI training dataset used for the 5-folds cross-validation.

|  | **A4-Learn** | **ADNI** | **ADNIDOD** | **HABS-HD** | **AMYPAD** | **AMYPAD** |
| --- | --- | --- | --- | --- | --- | --- |
|  | **FBP** | **FBP/FBB** | **FBP** | **FBB** | **FBB** | **FMM** |
| ***CL_Std_*** | 54.2 (39-59) | 25.0 (5-33) | 14.2 (12-22) | 62.7 (54-65) | 31.7 (25-46) | 20.0 (15-26) |
| ***CL_Comp_*** | 55.9 (54-59) | 10.3 (10-30) | 7.1 (-3-11)^**^ | - | - | - |
| ***CL_NMF_*** | 45.9 (43-51) | 13.4 (13-24) | 6.3 (1-10)^**^ | 59.0 (46-66) | 24.9 (21-40) | 18.0 (12-19) |
| ***CL_DS mask_*** | 57.3 (47-58) | 14.6 (15-38) | 11.2 (6-16) | 68.2 (57-74) | 30.3 (29-46) | 17.4 (11-20) |
| ***CL_DS_*** | 50.0 (50-55) | 18.5 (19-30) | 11.1 (8-16) | 60.9 (57-75) | 41.0 (35-52) | 13.1 (13-21) |

Sup Table 4. Centiloid thresholds (confidence interval) obtained using each quantification method maximising F1-score index AUC with visual reads in ADNI, ADNI-DOD, A4 and HABS-HD. For each threshold, the confidence interval is based on 10000 bootstraps. Where the threshold is significantly different compared to using CL_Std_, the significance is denoted using: **p<0.01

|  | MMSE vs CL (*ρ*) | CDR 0 vs CDR 0.5 (Cohen's *d*) | CDR 0.5 vs CDR 1 (Cohen's *d*) | CDR 0 vs CDR 1 (Cohen's *d*) |
| --- | --- | --- | --- | --- |
| *CL_Std_* | -0.077 | 0.272 | 0.644 | 0.960 |
| *CL_Comp_* | -0.077 | 0.244 | 0.691^***^ | 0.983 |
| *CL_NMF_* | -0.073 | 0.256 | 0.706^**^ | 1.017^**^ |
| *CL_DS mask_* | -0.093^***^ | 0.285 | **0.727**^***^ | 1.055^***^ |
| *CL_DS_* | **-0.111**^***^ | **0.303**^***^ | 0.718^***^ | **1.061**^***^ |

Sup Table 5. Correlation (Spearman rank) between the baseline CL and MMSE, and effect size when comparing CDR 0 to CDR 0.5, CDR 0.5 to CDR 1 and CDR 0 to CDR 1 in the external testing cohorts: A4-Learn (FBP=12.2%); ADNI (FBB=2.5%,FBP=9.1%,PIB=0.6%); ADNIDOD (FBP=1.6%); ADNeT (NAV=2.9%); AIBL (FBB=0.1%,FBP=3.2%,FMM=1.7%,NAV=2.3%,PIB=2.8%); HABS-HD (FBB=13.7%); MCSA (PIB=12.2%); OASIS (FBP=1.3%,PIB=3.7%). The strongest correlation and largest effect sizes are highlighted with bold font. For each method where the correlations and effect size are significantly improved compared to using CL_Std_, the significance is denoted using: * p<0.05, **p<0.01, *** p<0.001

|  | MMSE vs CL (*ρ*) | CDR 0 vs CDR 0.5 (Cohen's *d*) | CDR 0.5 vs CDR 1 (Cohen's *d*) | CDR 0 vs CDR 1 (Cohen's *d*) |
| --- | --- | --- | --- | --- |
| *CL_Std_* | -0.360 | 0.762 | 0.582 | 1.478 |
| *CL_Comp_* | -0.373 | 0.799^**^ | 0.686^***^ | 1.647^***^ |
| *CL_NMF_* | -0.380^**^ | 0.796^**^ | 0.686^***^ | 1.649^***^ |
| *CL_DS mask_* | -0.378^***^ | **0.804**^***^ | **0.710**^***^ | 1.706^***^ |
| *CL_DS_* | **-0.383**^***^ | 0.803^***^ | **0.710**^***^ | **1.708**^***^ |

Sup Table 6. Correlation (Spearman rank) between the baseline CL and MMSE, and effect size when comparing CDR 0 to CDR 0.5, CDR 0.5 to CDR 1 and CDR 0 to CDR 1 in training cohorts: ADNI (FBB=11.3%,FBP=40.5%,PIB=2.9%); AIBL (FBB=0.5%,FBP=14.3%,FMM=7.7%,NAV=10.3%,PIB=12.5%). The strongest correlation and largest effect sizes are highlighted with bold font. For each method where the correlations and effect size are significantly improved compared to using CL_Std_, the significance is denoted using: * p<0.05, **p<0.01, *** p<0.001

|  | Emerging Aβ+ (N/Total) (%) | | | | |
| --- | --- | --- | --- | --- | --- |
| Baseline CL | *CL_Std_* | *CL_Comp_* | *CL_NMF_* | *CL_DS-Mask_* | *CL_DS_* |
| [25-30] | 29/36 (81%) | 19/20 (95%) | **38/38 (100%)** | 31/32 (97%) | 31/32 (97%) |
| [20-25] | 23/39 (59%) | 27/30 (90%) | 27/31 (87%) | 32/36 (89%) | **31/31 (100%)** |
| [15-20] | 29/42 (69%) | 23/32 (72%) | 28/37 (76%) | 21/26 (81%) | **30/33 (91%)** |
| [10-15] | 34/50 (68%) | 32/53 (60%) | **29/41 (71%)** | 38/69 (55%) | 34/62 (55%) |
| [5-10] | **44/108 (41%)** | 39/100 (39%) | 27/82 (33%) | 23/127 (18%) | 29/114 (25%) |

Sup Table 7. Number of participants in the testing cohorts with emerging Aβ+ for each baseline CL bracket, and their proportion compared to the total number of participants within those brackets. For each bracket, the quantification methods with the highest proportion emerging Aβ+ are highlighted with bold font.

|  | Emerging Aβ+ (N/Total) (%) | | | | |
| --- | --- | --- | --- | --- | --- |
| Baseline CL | *CL_Std_* | *CL_Comp_* | *CL_NMF_* | *CL_DS-Mask_* | *CL_DS_* |
| [25-30] | **16/16 (100%)** | **13/13 (100%)** | **9/9 (100%)** | **12/12 (100%)** | **10/10 (100%)** |
| [20-25] | 23/30 (77%) | 23/27 (85%) | **18/18 (100%)** | **18/18 (100%)** | **9/9 (100%)** |
| [15-20] | 22/46 (48%) | 12/37 (32%) | 19/28 (68%) | **21/25 (84%)** | 21/26 (81%) |
| [10-15] | 24/54 (44%) | 25/50 (50%) | 26/47 (55%) | 23/41 (56%) | **25/33 (76%)** |
| [5-10] | 30/119 (25%) | 27/89 (30%) | 29/98 (30%) | 28/88 (31%) | **33/95 (35%)** |

Sup Table 8. Number of participants in the training cohorts with emerging Aβ+ for each baseline CL bracket, and their proportion compared to the total number of participants within those brackets. For each bracket, the quantification methods with the highest proportion emerging Aβ+ are highlighted with bold font.

|  | Slope | Intercept |
| --- | --- | --- |
| *PIB* | 0.633 | 0.886 |
| *FBP* | 0.336 | 0.878 |
| *FMM* | 0.470 | 0.860 |
| *NAV* | 0.685 | 0.878 |
| *FBB* | 0.409 | 0.852 |

Sup Table 9. Centiloid transforms for the DeepSUVR-derived masks. These are defined for each tracer in the same format as the standard Centiloid transform, so that CL_DS mask_ =100*(SUVR_DS mask_-Intercept)/Slope

|  | A4-Learn | ADNI | ADNIDOD | HABS-HD | AMYPAD | AMYPAD |
| --- | --- | --- | --- | --- | --- | --- |
| *CL_Std_* | 0.919 | 0.973 | 0.915 | 0.989 | 0.975 | 0.961 |
| *CL_Comp_* | 0.951^***^ | 0.965 | 0.927 | 0.989 | 0.975 | 0.961 |
| *CL_NMF_* | 0.948^***^ | 0.972 | 0.928 | 0.993^**^ | 0.975 | 0.973^***^ |
| *CL_DS mask_* | 0.951^***^ | 0.976 | **0.953^*^** | 0.994^***^ | 0.979 | 0.980^***^ |
| *CL_DS_* | **0.954**^***^ | **0.978** | 0.942^*^ | **0.994**^***^ | **0.980** | **0.981**^***^ |
| Tracers | FBP | FBP/FBB | FBP | FBB | FBB | FMM |
| N | 1772 | 80/58 | 230 | 1094 | 985 | 1275 |
| Visual Positivity (%) | 35.0 | 48.6 | 30.9 | 7.8 | 19.1 | 23.8 |

Sup Table10. AUC between visual reads in ADNI, ADNI-DOD, A4 and HABS-HD and the CL obtained using each quantification method. The quantification with the highest AUC for each cohort is presented using bold font. The quantifications highest AUC for each cohort is presented using bold font. The number of scans with visual reads, the tracer used, as well as the proportion of scan visually read as positive is presented under the Visual reads sub-heading. For each method where the AUC is significantly increased compared to using CL_Std_, the significance is denoted using: * p<0.05, **p<0.01, *** p<0.001

|  | AIBL/ADNI | | External | |
| --- | --- | --- | --- | --- |
|  | Neg outliers (%) | Pos outliers (%) | Neg outliers (%) | Pos outliers (%) |
| *CL_Std_* | 6.45 | 6.74 | 2.48 | 1.76 |
| *CL_Comp_* | 5.84 | 4.95 | 1.80 | 1.26 |
| *CL_NMF_* | 4.70 | 3.69 | 1.17 | 1.22 |
| *CL_DS mask_* | 2.11 | 2.86 | 0.52 | 0.56 |
| *CL_DS_* | **1.58** | **1.92** | **0.48** | **0.50** |

Sup Table 11. Percentage of outliers based on the 95% confidence interval of yearly change in CL_Std_ measured in PiB in AIBL, when no change of tracer or scanner occurred. The smallest percentage of outliers are presented using a bold font.
